# Reprogramming astroglia into neurons with hallmarks of fast-spiking parvalbumin-positive interneurons by phospho-site–deficient Ascl1

**DOI:** 10.1126/sciadv.adl5935

**Published:** 2024-10-25

**Authors:** Nicolás Marichal, Sophie Péron, Ana Beltrán Arranz, Chiara Galante, Franciele Franco Scarante, Rebecca Wiffen, Carol Schuurmans, Marisa Karow, Sergio Gascón, Benedikt Berninger

**Affiliations:** ^1^ Centre for Developmental Neurobiology, Institute of Psychiatry, Psychology & Neuroscience, King’s College London, London, UK.; ^2^ Institute of Physiological Chemistry, University Medical Center Johannes Gutenberg University, Mainz, Germany.; ^3^ Department of Pharmacology, Ribeirão Preto Medical School, University of São Paulo, São Paulo, Brazil.; ^4^ Biological Sciences Platform, Sunnybrook Research Institute, Toronto, ON, Canada.; ^5^ Department of Biochemistry, University of Toronto, Toronto, ON, Canada.; ^6^ Department of Laboratory Medicine and Pathobiology, University of Toronto, Toronto, ON, Canada.; ^7^ Institute of Biochemistry, Friedrich-Alexander-Universität Erlangen-Nürnberg, Erlangen, Germany.; ^8^ Department of Molecular, Cellular and Developmental Neurobiology, Cajal Institute – CSIC, Madrid, Spain.; ^9^ MRC Centre for Neurodevelopmental Disorders, Institute of Psychiatry, Psychology & Neuroscience, King’s College London, London, UK.; ^10^ The Francis Crick Institute, London, UK.; ^11^ Focus Program Translational Neuroscience, Johannes Gutenberg University, Mainz, Germany.

## Abstract

Cellular reprogramming of mammalian glia to an induced neuronal fate holds the potential for restoring diseased brain circuits. While the proneural factor *achaete-scute complex-like 1* (*Ascl1*) is widely used for neuronal reprogramming, in the early postnatal mouse cortex, *Ascl1* fails to induce the glia-to-neuron conversion, instead promoting the proliferation of oligodendrocyte progenitor cells (OPC). Since Ascl1 activity is posttranslationally regulated, here, we investigated the consequences of mutating six serine phospho-acceptor sites to alanine (Ascl1SA6) on lineage reprogramming in vivo. Ascl1SA6 exhibited increased neurogenic activity in the glia of the early postnatal mouse cortex, an effect enhanced by coexpression of *B cell lymphoma 2* (*Bcl2*). Genetic fate-mapping revealed that most induced neurons originated from astrocytes, while only a few derived from OPCs. Many Ascl1SA6/Bcl2-induced neurons expressed parvalbumin and were capable of high-frequency action potential firing. Our study demonstrates the authentic conversion of astroglia into neurons featuring subclass hallmarks of cortical interneurons, advancing our scope of engineering neuronal fates in the brain.

## Teaser

Mouse cortical glia can be converted into neurons featuring hallmarks of an important subclass of cortical interneurons.

## INTRODUCTION

Changing the identity of a terminally differentiated cell into another desired cell type via lineage reprogramming is an emerging experimental strategy for tissue repair in organs with little or no endogenous stem cell activity (*1*). In the context of the central nervous system, the generation of induced neurons (iNs) from other resident cell types, such as glial cells, opens new avenues for the remodeling and restoration of diseased brain circuits (*2*). Following pioneering studies demonstrating the possibility of generating iNs from primary glia in vitro (*3*, *4*), proof-of-principle studies have since provided evidence for glia-to-neuron conversion in various brain areas in vivo through ectopic expression of proneural transcription factors (*5*–*9*). More recently, however, important concerns about the authenticity and efficiency of in vivo glia-to-neuron conversion have been raised since many studies lacked effective lineage tracing to demonstrate the glial origin of iNs (*10*–*12*). Subsequent efforts that scrutinized the origin of putative iNs uncovered inadvertent labeling of endogenous neurons that could not be traced back to a glial origin (*10*). This emphasizes the importance of robust genetic fate mapping to unambiguously determine the glial origin of iNs (*13*–*15*).

The basic helix-loop-helix transcription factor achaete-scute complex-like 1 (Ascl1) is a key regulator of neural fate decisions ranging from cellular proliferation and cell cycle exit to neural fate specification in the embryonic and adult nervous system (*16*–*20*). Given its neurogenic activity, *Ascl1* has been widely and successfully used for converting somatic cells such as fibroblasts, glia, and other cell types into iNs in vitro, either alone or in combination with other transcription factors (*4*, *21*, *22*). However, the neurogenic activity of Ascl1 is highly cell and context dependent. For instance, on its own, Ascl1 failed to convert glia in the early postnatal cortex into neurons in vivo and instead promoted the proliferation of oligodendrocyte progenitor cells (OPCs) (*23*). Likewise, when expressed without other factors, *Ascl1* reprogrammed reactive glia in the lesioned adult cortex or the epileptic hippocampus into iNs only inefficiently (*5*, *7*). While several studies have highlighted the capacity of Ascl1 to act as a pioneer transcription factor (*24*, *25*), the high degree of variability of Ascl1 effects in vivo is suggestive of additional layers of regulation that may hinder its neurogenic activity. Ascl1 activity is regulated by posttranslational modifications, including phosphorylation (*26*–*29*). Preventing phosphorylation-dependent regulation of Ascl1 activity by mutating serine residues of six serine-proline motifs to alanine (Ascl1SA6) has been found to increase its neurogenic activity in the embryonic cerebral cortex (*26*). This finding has led us to hypothesize that using the *Ascl1SA6* mutant variant as a reprogramming factor could enhance glia-to-neuron conversion in vivo.

Here, we demonstrate that retroviral-mediated overexpression of *Ascl1SA6* in glia undergoing proliferative expansion in the early postnatal mouse cerebral cortex has enhanced neurogenic capacity compared to that of wild-type (wt) *Ascl1* in vivo. Furthermore, we found that coexpression of *Ascl1SA6* with *B cell lymphoma 2* (*Bcl2*), previously demonstrated to enhance iN survival (*6*), can more efficiently reprogram postnatal cortical glia into iNs. Through genetic fate mapping, we show that iNs originate predominantly from astrocytes and to a much lesser degree from OPCs. We also found that Ascl1SA6/Bcl2-iNs frequently exhibit hallmarks of fast-spiking (FS) parvalbumin (PV)-positive interneurons, an important subclass of γ-aminobutyric acid–releasing (GABAergic) interneurons in the mammalian cerebral cortex.

## RESULTS

### Enhanced in vivo neuronal reprogramming activity by a phospho-site mutant of Ascl1

We have recently established an experimental model to explore glia-to-neuron conversion in the early postnatal mouse cerebral cortex (*23*, *30*), in which MMLV (Moloney murine leukemia virus) retroviruses enable efficient targeting of both astrocytes and OPCs undergoing proliferative expansion (*31*, *32*). Given that we had previously observed that *Ascl1* failed to induce substantial neurogenesis (*23*), here, we wondered whether using a more neurogenic phospho-site mutant Ascl1 (Ascl1SA6) (*26*, *27*), in which six serine-proline sites had been altered to alanine-proline exhibited improved neuronal reprogramming (fig. S1A). To address this question, we compared the effects of *Ascl1*- or *Ascl1SA6*-encoding retroviruses upon injection in the mouse cerebral cortex at postnatal day 5 (P5). When analyzed at 12 days post injection (dpi), a time point when previous studies had observed successful glia-to-neuron conversion by other neurogenic transcription factors (*5*, *6*, *30*), we found that *Ascl1* elicited very limited expression of immature and mature neuronal markers doublecortin (Dcx) and neuronal nuclei (NeuN), respectively, in transduced cells, which mostly retained glial morphology (fig. S1, B and C). In sharp contrast, MMLV retrovirus–mediated expression of *Ascl1SA6* induced neuronal marker expression (Dcx, NeuN, or both) in almost half of the transduced cells (fig. S1, B and C). These data suggest that the activity of Ascl1 in proliferative glia is subject to posttranslational regulation and that the phospho-site mutant is more powerful in inducing neuronal fate conversion from glia than wt Ascl1.

**Fig. 1.**
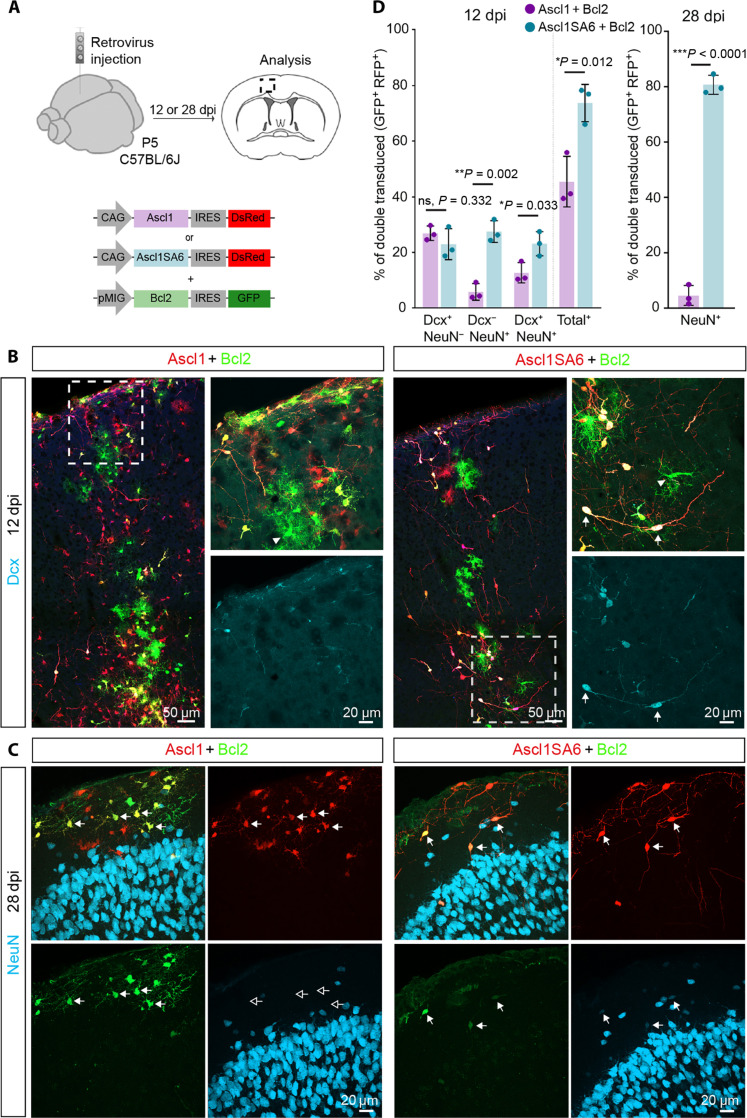
Coexpression of *Ascl1SA6* and *Bcl2* efficiently reprograms postnatal cortical glia into iNs. (**A**) Experimental design. Retroviral constructs encoding for *Ascl1* or *Ascl1SA6* and *Bcl2* were injected in the cortex of wt (C57BL/6J) mice at P5. Immunohistochemical analysis was performed at 12 or 28 dpi. (**B**) Low-magnification image showing *Ascl1-* or *Ascl1SA6/Bcl2*-transduced cells at 12 dpi. High-magnification images from insets (white boxes) depicting Dcx expression in cotransduced cells. Notice the acquisition of conspicuous neuronal morphology in Ascl1SA6/Bcl2 iNs (arrows). Cells transduced with *Bcl2* only (GFP^+^ only) retained glial morphology (arrowheads). (**C**) At 28 dpi, the vast majority of Ascl1SA6/Bcl2 iNs expressed NeuN, while Ascl1/Bcl2 iNs did not express this neuronal marker and maintained glial morphology. Empty arrows indicate marker-negative cells. (**D**) Proportion of double-transduced cells expressing Dcx, NeuN, or both neuronal markers at 12 dpi (left graph), and double-transduced cells expressing NeuN (right graph) at 28 dpi. 26.9 ± 2.6% Dcx only, 5.8 ± 3% NeuN only, 12.7 ± 3.6% Dcx and NeuN, 2380 cells, *n* = 3 mice for *Ascl1/Bcl2*; 23 ± 5.5% Dcx only, 27.5 ± 3.9% NeuN only, 23.2 ± 4.3% Dcx and NeuN, 836 cells, *n* = 3 animals for *Ascl1SA6/Bcl2* at 12 dpi. 0.0 ± 0.0% Dcx, 784 cells, *n* = 3 mice for *Ascl1/Bcl2*; 0.0 ± 0.0% Dcx, 286 cells, *n* = 3 mice for *Ascl1SA6/Bcl2* at 28 dpi; 4.5 ± 3.6% NeuN, 499 cells, *n* = 3 mice for *Ascl1/Bcl2*; 80.7 ± 3.5% NeuN, 157 cells, *n* = 3 mice for *Ascl1SA6/Bcl2* at 28 dpi. Data are shown as means ± SD. Two-tailed Student’s unpaired *t* test in (D).

Glial cells undergoing reprogramming by forced expression of proneural transcription factors are susceptible to cell death via ferroptosis, which can be mitigated by coexpression of *Bcl2* (*6*). Thus, we next examined the effect of *Bcl2* coexpression alongside *Ascl1* or *Ascl1SA6* (Fig. 1A). Cells transduced with *Bcl2* alone (green fluorescent protein–positive, GFP^+^) retained glial identity (Fig. 1B). While *Ascl1* expression alone showed negligible lineage conversion (fig. S1A), when coexpressed with *Bcl2*, we observed a substantial neurogenic response, as determined by Dcx and NeuN immunoreactivity in double-transduced cells (GFP^+^/red fluorescent protein–positive, RFP^+^) at 12 dpi (Fig. 1, B and D, and fig. S2, A and B). Neuronal fate conversion was further enhanced when *Bcl2* was coexpressed with *Ascl1SA6*, resulting in higher levels of NeuN expression and the appearance of cells with clear neuronal morphologies (Fig. 1, B and D, and fig. S2, A and B). Twenty-eight days after retroviral injection (28 dpi), virtually none of the Ascl1/Bcl2 and Ascl1SA6/Bcl2 cells continued expressing Dcx (Fig. 1D and fig. S2C). The vast majority of Ascl1/Bcl2 cells had not acquired NeuN expression and, by and large, exhibited glial morphology (fig. S2C), potentially indicating that iNs observed at 12 dpi had either died or reverted to a glial fate. While Ascl1/Bcl2 cells were mostly negative for the astroglial lineage determinant SRY-Box Transcription Factor 9 (Sox9) (fig. S3, A and C) (*33*) indicating that few cells had remained or reacquired an astroglial fate, most expressed the oligodendroglial lineage determinant Sox10 (fig. S3, B and D) (*34*). Thus, despite an earlier boosting of the neurogenic activity of *Ascl1* by *Bcl2*, Dcx-positive iNs might have either died or returned to an oligodendroglial identity. The relative proportion of Dcx, NeuN, or Sox9-positive cells among Ascl1/Bcl2 cells may have been further reduced by the potential proliferative expansion of Ascl1/Bcl2-expressing OPCs, as previously shown for OPCs transduced by *Ascl1* alone (*23*). In sharp contrast, nearly 80% of the Ascl1SA6/Bcl2 cells expressed NeuN (Fig. 1, C and D), indicating that the phospho-site mutant Ascl1SA6 had exerted a more persistent neurogenic activity as compared to Ascl1 and that previously Dcx-positive iNs had further matured toward a NeuN-positive stage. Consistent with successful neuronal reprogramming, Ascl1SA6/Bcl2 cells had efficiently down-regulated either Sox9 or Sox10, indicating that the acquisition of a neuronal fate was accompanied by a long-lasting loss of glial fate determinants (fig. S3). However, as compared to 12 dpi, at 28 dpi, we noted an overall drop in Ascl1SA6/Bcl2 iNs, as reflected in overall lower cell counts (Fig. 1D).

Previous studies have shown that increased protein stability might contribute to the heightened neurogenic activity of phospho-site mutant Ascl1 (*26*, *27*). In line with this, we found overall higher levels of Ascl1 immunoreactivity in cells expressing Ascl1SA6 as compared to Ascl1 at 12 dpi (fig. S4, A and B). These differences were no longer discernible at 28 dpi (fig. S4, C and D). Moreover, Ascl1 expression levels were markedly reduced as compared to 12 dpi. This may suggest that higher levels of Ascl1 protein may be necessary to promote reprogramming success at an earlier stage but may not be required once reprogramming has become consolidated.

To ascertain the specificity of the MMLV retroviral vectors in stably transducing-only proliferating glial cells and rule out unintended labeling of endogenous postmitotic neurons (*10*), we injected thymidine analog bromodeoxyuridine (BrdU) to label proliferating cells on the day of, and day following, MMLV retrovirus injection (fig. S5A). As expected, we found that a large proportion of transduced cells were BrdU positive (fig. S5, B and C). We found that the vast majority (~80%) of retroviral transduced cells that had acquired a neuronal morphology were BrdU positive (fig. S5, B and C) and coexpressed NeuN (fig. S5D), indicating that iNs derived from cells that had proliferated during this stage of postnatal cortical development. We consistently observed that the level of the BrdU signal was lower in cells retaining glial morphology, possibly due to dilution of the BrdU signal as a result of continued proliferation (fig. S5, E to G). These data provide indirect evidence that lineage conversion from glia to neuron occurs in the absence of further rounds of cell divisions following the initial mitosis required for retroviral transduction, as iNs maintain significantly higher BrdU signal levels as compared to non-reprogrammed glia which may undergo additional rounds of cell division before becoming postmitotic (fig. S5, E to G).

### Astroglial origin of Ascl1SA6/Bcl2 iNs

**Fig. 2.**
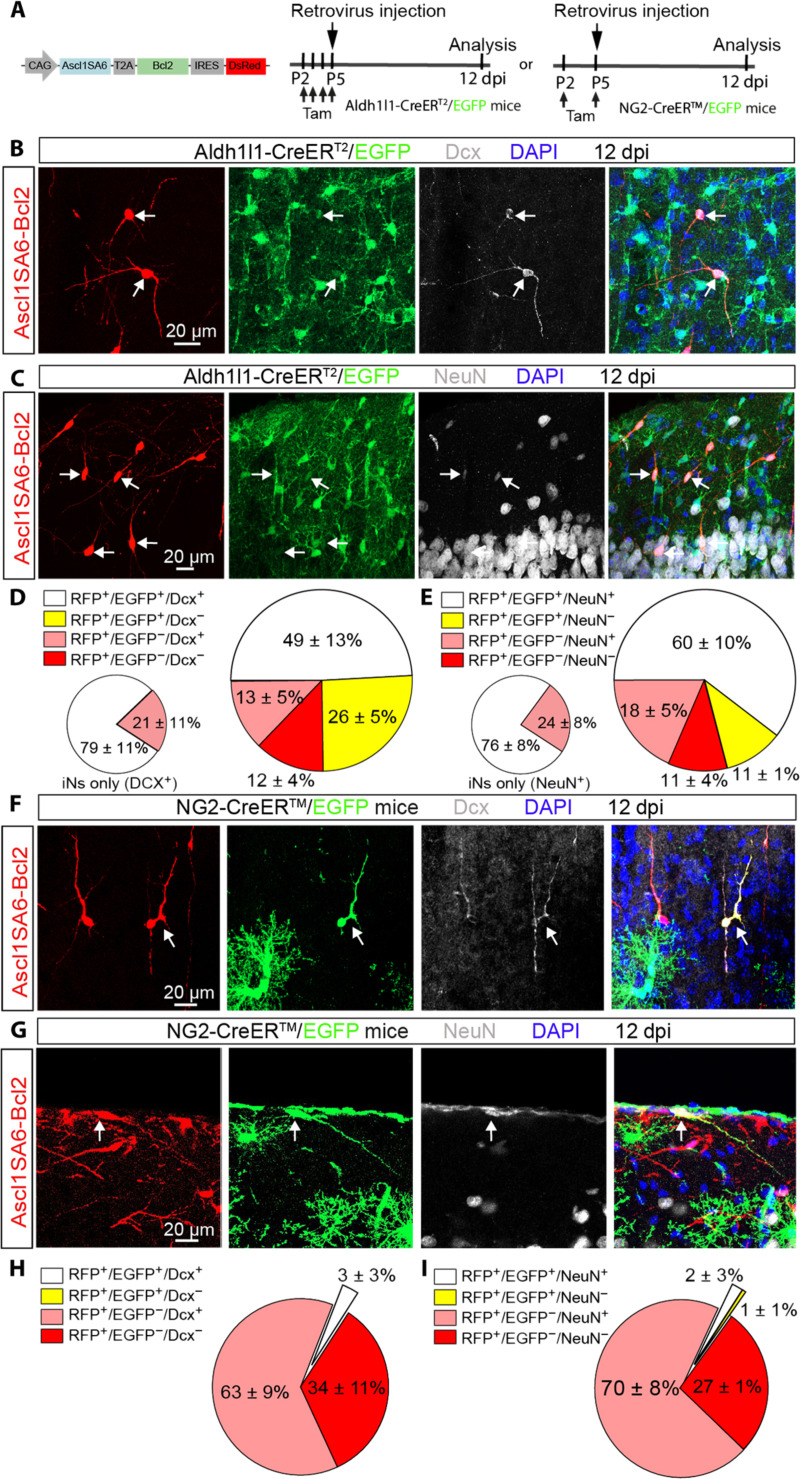
Astroglial origin of the vast majority of Ascl1SA6-Bcl2 iNs. (**A**) Experimental design. Retroviral constructs encoding for *Ascl1SA6-Bcl2* were injected in the cortex of *Aldh1l1-CreERT2/RCE:loxP* or *NG2-CreERTM/RCE:loxP* transgenic mice at P5. Mice received subcutaneous injection of tamoxifen before retroviral injection to induce Cre-mediated recombination and achieve irreversible labeling of astrocytes or oligodendrocyte precursor cells with EGFP. The proportion of cells with astroglial or oligodendroglial origin was analyzed at 12 dpi. (**B** and **C**) Ascl1SA6-Bcl2 iNs expressing Dcx (B) or NeuN (C) and GFP (arrows) in the *Aldh1l1-CreERT2/RCE:loxP* mice, demonstrating their astroglial origin. (**D** and **E**) Large pie charts showing the relative number of transduced cells (DsRed^+^) coexpressing EGFP and/or Dcx (D) and/or NeuN (E), or no other marker than the DsRed in *Aldh1l1-CreERT2/RCE:loxP* mice at 12 dpi. 523 cells, *n* = 3 mice for Dcx analysis; 473 cells, *n* = 3 mice for NeuN analysis. Small pie charts include only those DsRed^+^ cells that also expressed a neuronal marker (Dcx or NeuN); white sections, lineage traced (EGFP^+^); pink sections, no lineage tracing (EGFP^−^). (**F** and **G**) Ascl1SA6-Bcl2 iNs coexpressing Dcx (F) or NeuN (G) as well as the reporter genes *DsRed* and *EGFP* in *NG2-CreERTM/RCE:loxP* mice at 12 dpi. (**H** and **I**) Pie charts showing the relative number of transduced cells (DsRed^+^) coexpressing EGFP and/or Dcx (H) and/or NeuN (I), or no other marker than DsRed in *NG2-CreERTM/RCE:loxP* mice at 12 dpi. 416 cells, *n* = 3 mice for Dcx analysis; 169 cells, *n* = 3 mice for NeuN analysis. Data are shown as means ± SD.

Several glial cell types undergo local proliferation in the early postnatal cortex and could, therefore, be targeted by our retroviral constructs (*31*, *32*, *35*), raising the question of the cellular origins of iNs following *Ascl1SA6*- and *Bcl2*-mediated lineage conversion in the early postnatal cortex. To address this question, we combined reprogramming with *Ascl1SA6* and *Bcl2* together with genetic fate mapping. Astrocytes were fate mapped using *aldehyde dehydrogenase 1 family member L1-CreERT2/RCE:loxP* (*Aldh1l1-CreERT2/RCE:loxP*) transgenic mice (*36*), in which Cre-dependent expression of CMV-IE enhancer/chicken beta-actin/rabbit beta-globin (CAG) hybrid promoter-boosted enhanced GFP (EGFP) was induced by administration of tamoxifen in the days preceding viral injection (Fig. 2A). To allow for tracing EGFP-positive cells expressing the two reprogramming factors, we designed a single retroviral vector encoding for both *Ascl1SA6* and *Bcl2* (*Ascl1SA6-Bcl2*) and the *Discosoma sp. Red fluorescent protein* (*DsRed*) reporter (Fig. 2A), which exhibited a similar reprogramming efficacy as the individual vectors when injected in wt mice (fig. S6, A to C). Tamoxifen-treated *Aldh1l1-CreERT2/RCE:loxP* mice were injected with the *Ascl1SA6-Bcl2*–encoding retrovirus at P5, and the identity of fate-mapped cells was analyzed by immunostaining for GFP (identifying cells of astroglial origin), DsRed (identifying transduced cells), and Dcx or NeuN (identifying iNs) at 12 dpi (Fig. 2A). We found that more than two-thirds of the DsRed-positive transduced cells also expressed EGFP (i.e., retrovirus-targeted cells of astroglial origin; Fig. 2, B to E, white and yellow sections, large pie charts). Of these transduced astroglial cells, a high proportion was immunoreactive for Dcx (Fig. 2, B and D) and NeuN (Fig. 2, C and E). Of all cells classified as iNs (Fig. 2, D and E, white and pink sections, large pie charts), 80% could be lineage-traced to astrocytes (Fig. 2, D and E, white sections, small pie charts). The fact that, in nontransduced controls, virtually all EGFP^+^ cells coexpressed Sox9 but not Dcx or NeuN (fig. S7, A and B), demonstrated that tamoxifen administration at P2 to P5 had selectively labeled astrocytes without targeting any intrinsically neurogenic progenitors either of cortical or other forebrain origins (*37*). Likewise, no Dcx-positive cells were detected in the non-injected hemisphere contralateral to the site of Ascl1SA6/Bcl2-induced reprogramming (fig. S7C). Last, no neuronal marker–expressing cells could be fate mapped in control virus-injected, tamoxifen-induced *Aldh1l1-CreERT2/RCE:loxP* mice (fig. S7, D and E), further corroborating the astroglial origin of putative iNs and ruling out substantial contributions from residual neurogenic radial glia or other endogenous neuronal progenitors to the overall iN yield.

To corroborate an astroglial origin of Ascl1SA6/Bcl2 iNs, we performed lineage tracing in mouse *Glial fibrillary acidic protein-Cre/RCE:loxP* (*mGFAP-Cre/RCE:loxP*) mice in which astrocytes are stably labeled with EGFP (*38*). Despite a reduced efficiency of astrocyte fate mapping in this mouse line (fig. S8, A and B), we nevertheless found that a very substantial proportion of Dcx-positive and NeuN-positive transduced cells was derived from EGFP-positive astrocytes (fig. S8, C to G).

OPCs also sustain high proliferation in the postnatal cortex (*31*) and are transduced by retroviruses injected in the P5 postnatal cortex (*23*). We, therefore, tested next whether cells of the oligodendroglial lineage were also converted by *Ascl1SA6-Bcl2* into Dcx-positive or NeuN-positive iNs. To this end, genetic fate mapping was performed in *NG2-CreERTM/RCE:loxP* transgenic mice, in which cells with an active *Cspg4* locus encoding neuron-glial antigen 2 (NG2) are stably labeled by EGFP reporter expression upon tamoxifen injection (Fig. 2A) (*39*). Unexpectedly, we found that only approximately 3% of DsRed-positive transduced cells also expressed EGFP and Dcx or NeuN (Fig. 2, F to I), suggesting that a very small fraction of the Ascl1SA6-Bcl2 iNs were of oligodendroglial origin. To validate that MMLV retrovirus-transduced OPCs could be effectively lineage traced, we injected a control MMLV retrovirus (fig. S8H). We found that 22% of all transduced cells could be lineage traced to the oligodendroglial lineage (fig. S8, I and J), consistent with a previous characterization of MMLV retrovirus targeted glial population (*23*). As expected, transduced cells that were not fate mapped were mostly Sox9-positive astrocytes (fig. S8, I and J). Together, our genetic fate mapping experiments demonstrate the authenticity of *Ascl1SA6-Bcl2* glia-to-neuron lineage conversion in the early postnatal cortex, with astrocytes being the predominant cellular origins for observed iNs. Despite our retroviral vectors being able to successfully transduce cortical OPCs, these contribute only a very small fraction of iNs.

### Parvalbumin expression in Ascl1SA6/Bcl2 iNs

**Fig. 3.**
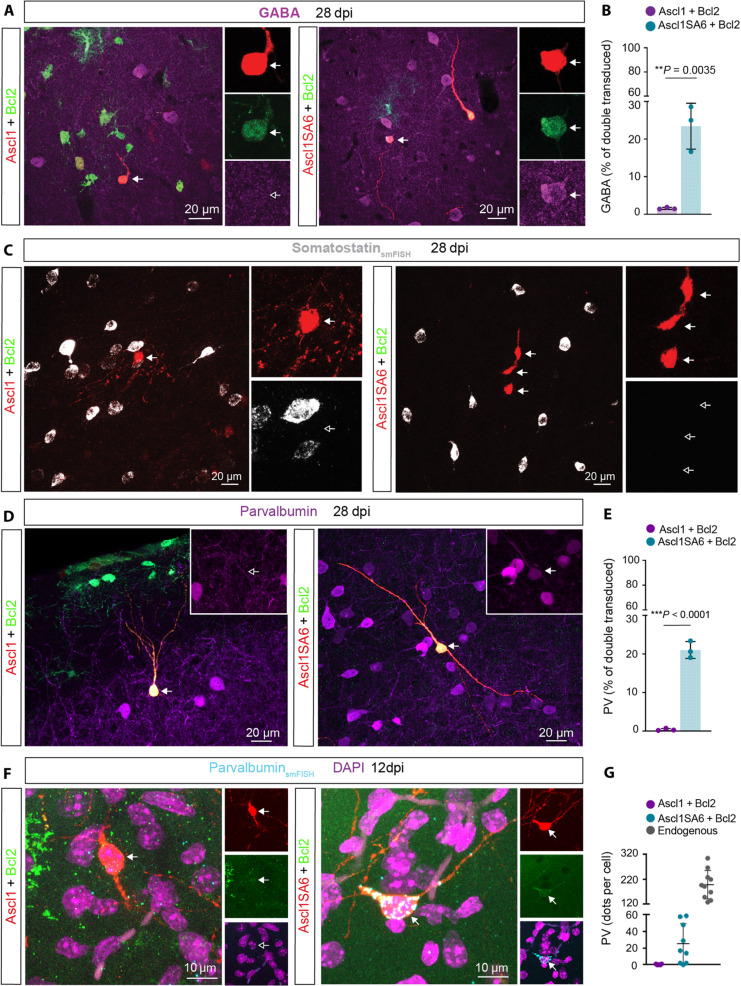
Forced coexpression of *Ascl1SA6* and *Bcl2* converts postnatal cortical glia into GABA- and PV-expressing iNs. (**A**) *Ascl1/Bcl2*-transduced cells do not express GABA (left), in contrast to the acquisition of GABA expression in Ascl1SA6/Bcl2 iNs (right), 28 dpi. Arrows indicate iNs lacking GABA (inset, empty arrow) or containing GABA (inset, filled arrow). (**B**) Proportion of double-transduced cells expressing GABA following *Ascl1/Bcl2* or *Ascl1SA6/Bcl2* reprogramming at 28 dpi. 1.6 ± 0.3%, 634 cells, *n* = 3 mice for *Ascl1/Bcl2*; 23.4 ± 6.1%, 78 cells, *n* = 3 mice for *Ascl1SA6/Bcl2*. Data are shown as means ± SD. (**C**) *Ascl1/Bcl2* and *Ascl1SA6/Bcl2* iNs lack *Sst* mRNA. Empty arrows highlight the lack of *Sst* expression in iNs (insets). (**D**) Analysis of PV expression in *Ascl1/Bcl2*-transduced cells (left) and Ascl1SA6/Bcl2 iNs at 28 dpi. Arrows indicate iNs lacking PV (inset, empty arrow) or containing PV (inset, filled arrow). (**E**) Proportion of double-transduced cells expressing PV at 28 dpi. 0.4 ± 0.3%, 2995 cells, *n* = 3 mice for *Ascl1/Bcl2*; 21.0 ± 2.2%, 208 cells, *n* = 3 mice for *Ascl1SA6/Bcl2*. Data are shown as means ± SD. (**F**) *Ascl1/Bcl2*-transduced cells (left) lack *Pvalb* mRNA, while Ascl1SA6/Bcl2 iNs (right) express *Pvalb* mRNA already at 12 dpi. Empty arrows indicate marker-negative cells. (**G**) Quantification of *Pvalb* mRNA transcripts expressed as the total number of dots detected per individual cell in *Ascl1/Bcl2*, *Ascl1SA6/Bcl2*-transduced cells, and endogenous surrounding PV interneurons. Each dot represents one cell. 7 cells, *n* = 3 mice for *Ascl1/Bcl2*; 9 cells, *n* = 3 mice for *Ascl1SA6/Bcl2* cells; 10 cells, *n* = 3 mice for endogenous neurons. Data are shown as means ± SD. Two-tailed Student’s unpaired *t* test in (B) and (E).

A central aim of glia-to-neuron conversion is to identify reprogramming factors that allow for the generation of neurons with subtype-specific features in vivo. Ascl1 is known to be a pioneer transcription factor involved in GABAergic neuronal fate specification at early embryonic stages (*40*–*42*). At 28 dpi, we detected GABA immunoreactivity in a very small fraction of Ascl1/Bcl2 iNs. In contrast, approximately 25% of Ascl1SA6/Bcl2 iNs expressed this marker (Fig. 3, A and B), consistent with the role of unphosphorylated Ascl1 in GABAergic neurogenesis (*26*). To further characterize these iNs, we next examined whether they had acquired hallmarks of main subclasses of cortical interneurons, such as expression of the neuropeptide somatostatin (SST) or the calcium-binding protein PV (*43*). At 28 dpi, *Sst* mRNA was found neither in Ascl1/Bcl2 iNs nor in AsclSA6/Bcl2 iNs (Fig. 3C). Likewise, virtually none of the *Ascl1/Bcl2* transduced cells expressed PV. However, approximately 20% of Ascl1SA6/Bcl2 iNs acquired PV immunoreactivity at 28 dpi (Fig. 3, D and E). We also observed the coexpression of PV and GABA in Ascl1SA6/Bcl2 iNs (fig. S10A). Furthermore, single-molecule fluorescence in situ hybridization (smFISH) revealed that Ascl1SA6/Bcl2 iNs expressed *Pvalb* mRNA already at 12 dpi (Fig. 3, F and G), albeit at lower levels than endogenous PV cortical interneurons (Fig. 3G). When induced and endogenous PV neurons were compared, we noted that the former exhibited a significantly smaller soma size, which was not different from those iNs not expressing PV (fig. S9, A and B). Most PV-positive iNs displayed unipolar or bipolar morphologies with different degrees of branching (fig. S9, C and D). Given the large percentage of iNs being negative for PV (and *Sst*), we tested whether Ascl1SA6/Bcl2 iNs exhibited molecular hallmarks of other cortical neuron subclasses. For this, we analyzed mRNA or protein expression for other subclasses of cortical interneurons, such as the *5-hydroxytryptamine (serotonin) receptor 3A* (*Htr3a*) and vasoactive intestinal polypeptide (VIP), and of cortical projection neurons, such as Satb2 and Ctip2. We found that few Ascl1SA6/Bcl2 iNs expressed *Htr3a* mRNA transcripts, albeit at low levels. In contrast, we did not detect VIP, Satb2, or Ctip2 (fig. S10, B to F). Together, these results show that joint overexpression of *Ascl1SA6* and *Bcl2* can induce the conversion of postnatal cortical astroglia into iNs expressing both GABA and PV but may fail to robustly induce hallmarks of other cortical neuron fates.

### Fast-spiking phenotype of Ascl1SA6/Bcl2 iNs

**Fig. 4.**
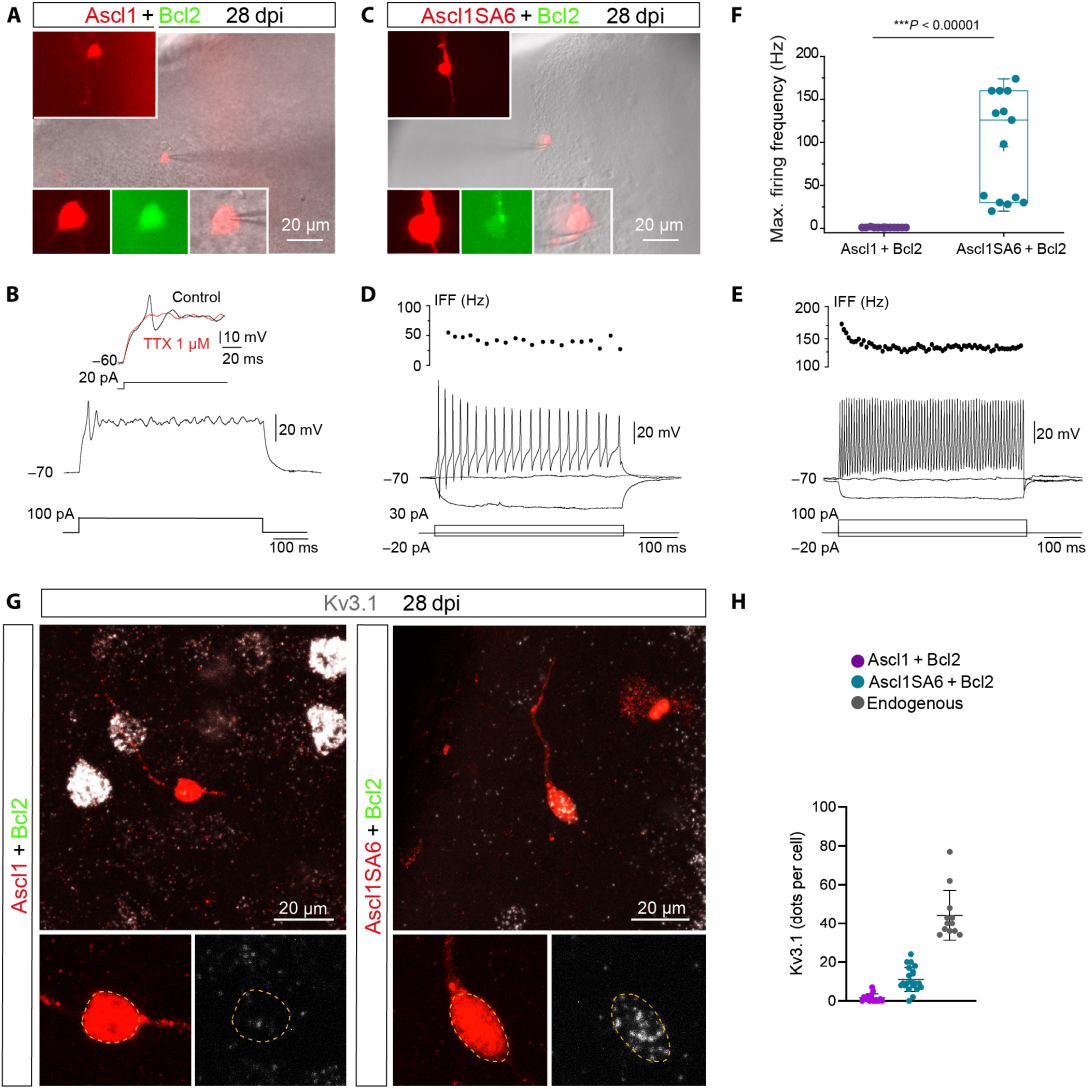
Induced neurons generated by coexpression of *Ascl1SA6* and *Bcl2* acquired functional properties of fast-spiking interneurons. (**A**) Example of recorded *Ascl1/Bcl2* cotransduced cell visualized in an acute brain slice. Insets show DsRed expression depicting the morphology of the cell (top inset) and colocalization of DsRed and GFP in the soma (bottom insets). (**B**) Ascl1/Bcl2 cell showing a single small spike in response to depolarizing current injection (12 of 13 recorded cells, *n* = 5 mice). The single spike was sensitive to tetrodotoxin (TTX) (three of three cells), indicating that it was mediated by voltage-gated Na^+^ channels. (**C**) Recorded *Ascl1SA6/Bcl2* cotransduced cell visualized in an acute brain slice. Insets similar as in (A). (**D**) Ascl1SA6/Bcl2 iN showing repetitive action potential with an instantaneous firing frequency (IFF) in the range of 50 Hz in response to depolarizing current injection (6 of 14 recorded cells, *n* = 8 mice). (**E**) Ascl1SA6/Bcl2 iN showing sustained high IFF (>100 Hz, 8 of 14 recorded cells, *n* = 8 mice). (**F**) Maximum firing frequency values in recorded Ascl1/Bcl2 and Ascl1SA6/Bcl2 iNs. Each dot represents one cell. Data are shown with box and whisker plots, which give the mean (+), median, 25th and 75th percentiles, and range. (**G**) iNs expressing mRNA of the voltage-gated K^+^ channel Kv3.1 (*Kcnc1*). (**H**) Quantification of *Kcnc1* mRNA transcripts expressed as the total number of dots detected in individual cells in *Ascl1/Bcl2*, *Ascl1SA6/Bcl2*-transduced cells, and endogenous surrounding interneurons. Each dot represents one cell. 15 cells, *n* = 3 mice for *Ascl1/Bcl2*-transduced cells; 21 cells, *n* = 3 mice for *Ascl1SA6/Bcl2*-transduced cells; 12 cells, *n* = 3 mice for endogenous neurons. Two-tailed Mann-Whitney test in (F).

PV-positive cortical interneurons show characteristic electrophysiological properties including FS firing (*43*, *44*). Given the expression of GABA and PV in Ascl1SA6/Bcl2 iNs, we next asked whether they acquired functional properties reminiscent of FS/PV interneurons by performing patch-clamp recordings in acute ex vivo cortical slices at 28 dpi (Fig. 4, A and C). While Ascl1/Bcl2 iNs mostly retained glial-like membrane properties, Ascl1SA6/Bcl2 iNs showed significantly higher input resistances and exhibited an overall tendency toward more negative resting membrane potentials (RMPs) and greater membrane capacitances, which were suggestive of an immature neuronal phenotype (fig. S12, A to C). Consistent with this, Ascl1/Bcl2 cells could generate only a single spikelet followed by non-regenerative membrane potential oscillations in response to sustained depolarization (Fig. 4B). This single spikelet was blocked by tetrodotoxin (TTX) (Fig. 4B), confirming that it was mediated by voltage-gated Na^+^ channels. Only one Ascl1/Bcl2 cell out of 13 cells recorded showed passive responses to depolarizing current pulses.

In sharp contrast, all Ascl1SA6/Bcl2 cells recorded (*n* = 14 cells) were able to repetitively fire well-developed action potentials in response to depolarization (Fig. 4, C to E). About half of them generated sustained trains of spikes with an instantaneous firing frequency (IFF) in the range of 50 Hz (Fig. 4D). The other half of the recorded cells exhibited sustained high-frequency firing, reaching frequencies above 150 Hz with a small drop in frequency firing during a period of constant activation (i.e., adaptation) (Fig. 4, E and F)—two distinctive features of endogenous cortical FS interneurons (*45*, *46*). Most Ascl1SA6/Bcl2 iNs exhibited a narrow action potential that included a well-developed afterhyperpolarization (AHP) in response to 10-ms somatic current injection (fig. S11A), resembling action potential waveforms of endogenous FS interneurons (*46*). As expected, the amplitude of the action potential and the AHP of Ascl1SA6/Bcl2 iNs were significantly higher, and the action potential half-width was significantly shorter than their counterparts in Ascl1/Bcl2 iNs (fig. S11, B to D). We also evaluated whether iNs became integrated into the host circuitry by monitoring spontaneous postsynaptic currents. Consistent with the immature functional phenotype of Ascl1/Bcl2 iNs, only a small proportion of these cells exhibited spontaneous synaptic currents (fig. S12, D, F, and G). In contrast, the vast majority of Ascl1SA6/Bcl2 iNs exhibited postsynaptic currents (fig. S12, E to G) that were blocked by the AMPA/kainate receptor blocker 6-cyano-7-nitroquinoxaline-2,3-dione (CNQX) (fig. S12H). While the number of iNs receiving synaptic input was fewer in the Ascl1/Bcl2 compared to the Ascl1SA6/Bcl2 group (Ascl1/Bcl2: 4/13 cells versus Ascl1SA6/Bcl2: 11/14 cells), the inputs did not differ significantly in frequency and amplitude between the two cohorts (fig. S12, F and G). Since we observed FS properties in iNs, we analyzed the expression of the delayed rectifying potassium channel Kv3.1 in iNs. Kv3.1 expression is mainly restricted to FS cortical interneurons and is critical for FS properties by providing rapid membrane repolarization after an action potential (*47*). Accordingly, we found that most of the Ascl1SA6/Bcl2 iNs expressed Kv3.1 (*Kcnc1*) mRNA and protein (Fig. 4G and fig. S11E), albeit at lower levels than endogenous interneurons (Fig. 4H and fig. S11E). Overall, these data demonstrate that *Ascl1SA6/Bcl2* can convert cortical glia into iNs that acquire functional hallmarks of FS interneurons.

### Induced fast-spiking– and parvalbumin-expressing iNs in cortical layer I

**Fig. 5.**
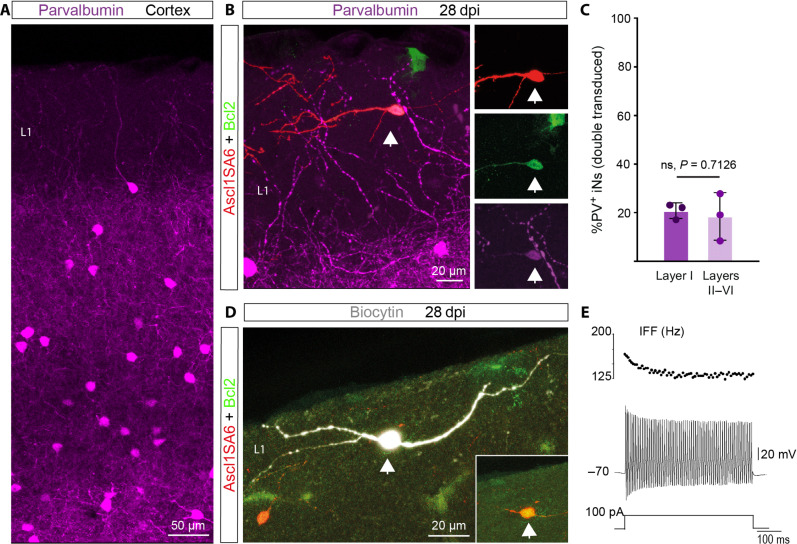
Fast-spiking– and parvalbumin-expressing iNs can be located in cortical layer I. (**A**) Expression of PV in the cerebral cortex. Note the absence of PV-positive interneurons in cortical layer I. (**B**) PV-positive Ascl1SA6/Bcl2 iN (arrow) generated in cortical layer I. (**C**) Quantification of the percentage of PV-positive iNs in layer I and the other layers (II to VI). 20.73 ± 3.2% in layer I, 18.39 ± 9.7% in layers II to VI. 161 PV^−^ iNs, 42 PV^+^ iNs, *n* = 3 mice. Data are shown as means ± SD. (**D**) Biocytin-filled Ascl1SA6/Bcl2 iN recorded in layer I. The inset shows the expression of the retroviral reporter proteins DsRed and GFP. (**E**) FS properties (IFF >100 Hz) observed in a Ascl1SA6/Bcl2 iN located in cortical layer I (six out of eight FS iNs). Two-tailed Student’s unpaired *t* test in (C).

In the mouse cortex, FS- and PV-expressing interneurons are located in layers II to VI but are absent from layer I (Fig. 5A) (*44*). Given that *Ascl1SA6/Bcl2* can induce the expression of PV in iNs, we next addressed whether these reprogramming factors could induce PV expression in an ectopic location. We found PV-positive Ascl1SA6/Bcl2 iNs are located in cortical layer I (Fig. 5, B and C). At 28 dpi, the relative proportion of PV-positive Ascl1SA6/Bcl2 iNs in layer I was similar to that found in deeper layers (layers II to VI), suggesting that PV-positive iNs are generated similarly across different layers of the cortex (Fig. 5C). Moreover, consistent with the presence of PV-positive iNs, we observed FS properties recorded from Ascl1SA6/Bcl2 iNs located in layer I (Fig. 5, D and E). These data suggest that overexpression of *Ascl1SA6* together with *Bcl2* can induce iNs with hallmarks of FS/PV interneurons independently of layer-specific instructive signals provided by the local environment.

## DISCUSSION

We show here that neuronal reprogramming of glia in the early postnatal cerebral cortex by the transcription factor Ascl1 is enhanced by using the phospho-site variant Ascl1SA6, in which six serine residues are mutated to alanine (*26*). Stringent genetic fate mapping showed that astrocytes are the main endogenous starting population of the iNs, while very few iNs are derived from OPCs. Moreover, overexpression of *Ascl1SA6* in combination with *Bcl2* (*6*) not only boosts the efficiency of glia-to-neuron conversion and/or iN survival but also generates iNs with specific histochemical and electrophysiological hallmarks of FS/PV-positive interneurons. Last, we show that FS- and PV-expressing iNs can be generated in an ectopic location in the cortex.

We provide compelling evidence that glia-to-neuron conversion is authentic: (i) Consistent with our experimental approach that aimed at selectively targeting glia undergoing proliferative expansion, we found that iNs effectively incorporated BrdU, when administered around the time of retrovirus injection; (ii) genetic fate mapping experiments demonstrated that approximately 80% of Ascl1SA6/Bcl2 iNs were lineage traced to astrocytes. While the proportion of astrocytes targeted by the *Aldh1l1-CreERT2* mouse line is very high (*36*), it does not capture all cortical astrocytes. It is thus plausible that some of the remaining 20% of nonlineage-traced iNs originated from astrocytes that failed to undergo recombination. Overall, these data argue strongly against unspecific labeling of endogenous neurons (*1*, *10*, *15*).

Our results demonstrate the important role of the phosphorylation state of Ascl1 in its neurogenic activity for glia-to-neuron conversion in vivo. The fact that the effects of mutant and wt *Ascl1* overexpression differ greatly strongly suggests that wt Ascl1 does become phosphorylated in postnatal cortical glia. This is in line with previous findings demonstrating the impact of phosphorylation on proneural factors, as well as studies using similar phospho-site–deficient constructs in progenitors during development and in human neuroblastoma cells (*20*, *26*–*28*). The enhanced neurogenic potential of the mutant variant reported here could be attributed to different mechanisms of action: (i) higher protein stability of Ascl1SA6 due to differences in ubiquitination (*48*) that might increase the efficiency to target binding sites with less accessibility, such as neuronal differentiation genes; we observed that at earlier stages of reprogramming *Ascl1SA6*-expressing cells exhibited significantly higher levels of Ascl1 protein than cells expressing wt *Ascl1*, and this could be a consequence of a reduced degradation rate; (ii) changes in binding properties, e.g., different binding partners by Ascl1SA6 compared to wt Ascl1. GSK3-mediated phosphorylation of the proneural factor Neurogenin 2 (Neurog2) promotes binding to *Lin*-11 *Isl*-1 *Mec*-3 homeodomain transcription factors in the embryonic spinal cord to induce a motor neuron fate (*49*); (iii) mutations might cause direct changes in protein structure, enhancing the pioneer activity of Ascl1SA6, especially given that phosphorylation adds a negative charge that might interfere with interactions with negatively charged DNA. Last, we also confirmed a synergistic effect of *Bcl2* to promote neuronal reprogramming and/or enhance the survival of iNs, consistent with previous reports in vitro and in vivo in combination with the proneural factor *Neurog2* (*6*).

Our results highlight the importance of cellular context for glia-to-neuron conversion. While our retroviral approach targets both proliferative astrocytes and OPCs (*23*), we found that astrocytes are more efficiently converted into iNs by *Ascl1SA6/Bcl2* compared to OPCs. Which subtypes of astrocytes (*50*) were converted into iNs and whether astrocyte heterogeneity may have an impact on reprogramming competence remains to be elucidated. Given that OPCs can be transduced by control retroviruses in a proportion of around 20% of the total transduced cells, it is intriguing that less than 3% of Ascl1SA6/Bcl2 iNs were OPC derived. One intriguing possibility may consist in differential phosphorylation of serine-150 in the second helix region of Ascl1, due to enhanced activity of a non-proline–directed serine/threonine kinase in OPCs as compared to astrocytes. Phosphorylation of this serine has been proposed as a general off switch for proneural factor activity (*51*). Alternatively, the neurogenic activity of Ascl1SA6 might induce enhanced cell death of OPCs despite retroviral *Bcl2* expression. The limited reprogramming competence of OPCs in the early postnatal cortex contrasts with the high reprogramming competence of OPCs previously observed in the injured adult brain and spinal cord (*5*, *52*, *53*).

A key finding of our study is the observation that *Ascl1SA6* promotes the emergence of FS phenotype and PV expression from glia in vivo, properties that are hallmarks of FS/PV^+^ GABAergic interneurons. Dysfunction of these interneurons is implicated in neuropsychiatric disorders (*54*, *55*). Notable efforts have been dedicated to generating this interneuron subtype from induced pluripotent stem cells for disease modeling and potential cell transplantation but has proved challenging (*56*–*58*). Our data show that around 20% of Ascl1SA6/Bcl2 iNs express PV protein and 50% of the recorded cells exhibited an FS phenotype. Although our reprogramming approach is unlikely to generate *bona fide* FS/PV^+^ interneurons, our results suggest that *Ascl1SA6/Bcl2* activates gene expression modules contributing to FS/PV identity, including the expression of the potassium channel Kv3.1. The activation of such modules can occur in an ectopic location, e.g., cortical layer I, suggesting that transcription factors can exert a determinant effect on the expression of interneuron subclass hallmarks irrespective of influences from the local environment. This does not exclude the possibility that cortical projection neurons could further refine iN properties and distribution, as occurs with endogenous interneurons (*59*).

While iNs matured from 12 days to 4 weeks as reflected by the decrease in Dcx and increase in NeuN expression, we also noted a reduction in iNs over time, likely reflecting substantial cell death. Studies in the injured adult hippocampus using very similar retroviral vectors observed survival of iNs over several months that could lead to improvement of diseased brain function such as suppression of seizure activity (*6*, *7*). Damaged brain circuits may be more conducive to the long-term incorporation of new neurons as compared to those that have not undergone overt damage (*60*). Thus, it will be important to understand the role of the microenvironment in promoting or limiting the survival of iNs to further enhance the functional impact iNs could exert on diseased brain circuits.

By using *Ascl1SA6* as a reprogramming factor, we succeeded in generating iNs with hallmarks of FS/PV-expressing interneurons from astrocytes in vivo. However, the retroviral approach used in this study necessarily limited neuronal reprogramming to glial cells undergoing active proliferation whose occurrence in the mouse brain is restricted to postnatal development and injury. Thus, future studies using alternative approaches will be required to uncover whether fully mature astrocytes in the uninjured brain exhibit similar reprogramming competence, and if not, whether such competence can be reinduced by additional molecular manipulations that allow for remodeling of a potentially more refractory epigenome of mature astrocytes. Our findings may thus pave the way toward leveraging lineage reprogramming of glia into subtype-specific neurons for restoration of diseased brain circuits.

## MATERIALS AND METHODS

### Plasmids and retroviruses

MMLV-based retroviral vectors (*61*) were produced to express *Ascl1* and *Ascl1SA6*, as previously described (*23*). Briefly, to generate the *pCAG-Ascl1-IRES-DsRed* and *pCAG-Ascl1SA6-IRES-DsRed* retroviral constructs, a cassette containing the coding sequences flanked by attL recombination sites was generated through the excision of the coding sequences for *Ascl1* and *Ascl1SA6* from pCIG2 parental vectors (*26*). We used the previously described construct for *Bcl2*: *pMIG_Bcl2_IRES_GFP* (*6*). We generated a retroviral backbone allowing for polycistronic expression of *Ascl1* or *Ascl1SA6* and *Bcl2* (connected via a T2A peptide sequence) together with DsRed, and under control of the CAG promoter: *pCAG-Ascl1-T2A-Bcl2-IRES-DsRed* and *RV-pCAG-Ascl1SA6-T2A-Bcl2-IRES-DsRed*. Viral particles were produced using gpg helper-free packaging cells to generate vesicular stomatitis virus glycoprotein–pseudotyped retroviral particles (*62*). Retroviral particles were harvested and concentrated from supernatants of transfected packaging cells by ultracentrifugation following standard protocols, resuspended in TBS (tris-buffered saline), and stored at −80°C until use. Viral titers used for experiments were in the range of 10^6^ to 10^8^ transducing units/ml.

### Animals and animal procedures

The study was performed in accordance with the guidelines of the German Animal Welfare Act, the European Directive 2010/63/EU for the protection of animals used for scientific purposes, and the Animal (Scientific Procedures) Act 1986 and was approved by local authorities (Rhineland-Palatinate State Authority, permit number 23 177 07-G15-1-031; ethical committee of King’s College London and the UK Home Office, permit numbers PD025E9BC and PP8849003). Mice were kept in a 12:12-hour light-dark cycle with food and water ad libitum. Male and female C57BL/6J pups were purchased with their mother from Janvier Labs (Le Genest-Saint-Isle, France) or bred in-house from adult mice purchased from Charles River Laboratories (Walden, UK). Male and female transgenic mice used in this study were generated in-house. For this purpose, mice in which the expression of Cre recombinase is driven by mouse *GFAP* promoter (*mGFAP-Cre*) [*B6.Cg-Tg(Gfap-cre)77.6Mvs/2 J*, JAX024098] (*38*) or in which tamoxifen-inducible Cre recombinase is driven by the *aldehyde dehydrogenase 1 family member L1* locus (*Aldh1l1*) (*Aldh1l1-Cre/ERT2*, JAX031008) (*36*) or the mouse NG2 promoter (*NG2-CreERTM*, JAX008538) (*39*) were crossed with an EGFP reporter mouse line (*RCE:loxP*, JAX032037) (*63*) to generate double transgenic animals (*mGFAP-Cre/RCE:loxP*, *Aldh1l1-Cre/ERT2/RCE:loxP*, or *NG2-CreERTM/RCE:loxP*). In *Aldh1l1-Cre/ERT2* or *NG2-CreERTM/RCE:loxP* mice, tamoxifen induction of Cre recombinase activity was performed by daily subcutaneous administration of tamoxifen to pups from P2 to P5 or at P2 and P5, respectively [60 μl each of a tamoxifen solution (6 mg/ml; ApexBio Technology, #B5965) prepared in corn oil (Sigma-Aldrich, Merck, Germany, #C8267) and 10% ethanol (EtOH)]. Retroviral injections targeted to the somatosensory and visual cortical areas were performed, as previously described (*23*). Briefly, pups at P5 were anesthetized and received a volume of 0.5 to 1 μl of retroviral suspension using glass capillaries through a small skull incision. The coordinates of reference used for injection were the following: +3 mm rostrocaudal from lambda, ±0.5 mm mesolateral from the midline, and −0.5 mm ventral. After injection, the wound was closed with surgical glue (3M Vetbond, NC0304169), and pups were left to recover on a warm plate/chamber (37°C) before returning them to their mother. The recovery state was checked daily for a week after the surgery.

### Immunohistochemistry

Tissue preparation and immunostainings were performed, as described previously (*23*). Briefly, animals were lethally anesthetized by intraperitoneal administration of ketamine (120 mg/kg; Zoetis) and xylazine (16 mg/kg; Bayer) or medetomidine (1 mg/kg; Orion Pharma), prepared in 0.9% NaCl, and transcardially perfused with 0.9% NaCl followed by 4% paraformaldehyde (PFA; Sigma-Aldrich, Merck, Germany, P6148). The brains were harvested and postfixed overnight in 4% PFA at 4°C. Then, 40-μm-thick coronal sections were prepared using a vibratome (Microm HM650V, Thermo Fisher Scientific, Waltham, MA, USA, or Leica VT1000S) and stored at −20°C in a cryoprotective solution containing 20% glucose (Sigma-Aldrich, Merck, Germany, G8270), 40% ethylene glycol (Sigma-Aldrich, Merck, Germany, 324558), and 0.025% sodium azide (Sigma-Aldrich, Merck, Germany, S2202). Using a free-floating procedure, brain sections were washed three times for 15 min with 1× TBS (50 mM tris, Invitrogen, 15504-020; 150 mM NaCl, Amresco, 0241; pH 7.6) and then incubated for 1.5 hours in blocking solution: 2.5% donkey serum (Sigma-Aldrich, Merck, Germany, S30), 2.5% goat serum (Sigma-Aldrich, Merck, Germany, S26), 0.3% Triton X-100, and 1× TBS. Slices were then incubated with primary antibodies diluted in blocking solution for 2 to 3 hours at RT, followed by overnight incubation at 4°C. After three washing steps with 1× TBS, slices were incubated with secondary antibodies diluted in blocking solution for 2 hours at RT. Slices were washed twice with 1× TBS, incubated with 5 μM 4′,6-diamidino-2-phenylindole (DAPI) in 1× TBS for 5 min at room temperature (RT), and washed three times with 1× TBS. For mounting, slices were washed two times with 1× phosphate buffer (PB; 30 mM Na_2_HPO_4_·12H_2_O, Merck, 10039-32-4; 33 mM NaH_2_PO_4_·2H_2_O, Merck, 13472-35-0; pH 7.4) and were dried on SuperFrost (Thermo Fisher Scientific, Waltham, MA, USA) microscope slides. Sections were further dehydrated with toluene and covered with cover glasses mounted with DPX mountant for histology (Sigma-Aldrich, Merck, Germany, 06522) or directly mounted with ProLong Gold (Thermo Fisher Scientific, Waltham, MA, USA, P36930) or with Mowiol (Polysciences, Warrington, PA, USA, #17951-500) supplemented with DABCO (#15154-500, Polysciences, Warrington, PA, USA). The following primary antibodies were used: anti-Ascl1 (mouse IgG1, 1:400; Franklin Lakes, NJ, USA, 556604), anti-Ctip2 (rat, 1:500; Abcam, Cambridge, UK, ab18465), anti-Dcx (goat, 1:250; Santa Cruz Biotechnology, Dallas, TX, USA, sc-8066), anti-Dcx (guinea pig, 1:500; Merck Millipore, Burlington, MA, USA, AB2253), anti-GABA (rabbit, 1:300; Sigma-Aldrich, Merck, Germany, A2052), anti-GFP (chicken, 1:1000; AvesLab, Davis, CA, USA, GFP-1020), anti-GFP (goat, 1:500; Abcam, Cambridge, UK, ab5450), anti-Kv3.1 (mouse IgG1, 1:400; NeuroMab, Davis, CA, USA, 75-041), anti-mCherry (chicken, 1:300; EnCor Biotechnology, Gainsville, FL, USA, CPCA-mCherry), anti-NeuN (mouse IgG1, 1:500; Merck Millipore, Burlington, MA, USA, MAB377), anti-PV (guinea pig, 1:1000; Synaptic Systems, Göttingen, Germany, 195004), anti-PV (mouse IgG1, 1:500; Sigma-Aldrich, Merck, Germany, P3088), anti-RFP (rabbit, 1:500; Biomol, Hamburg, Germany, 600401379S), anti-Sox9 (mouse IgG1, 1:400; eBiocience, San Diego, CA, USA, GMPR9), anti-Sox10 (goat, 1:300; Minneapolis, MN, USA, AF2864), anti-Satb2 (rat, 1:300; Abcam, Cambridge, UK, ab92446), and anti-VIP (rabbit, 1:1000; Immunostar, Hudson, WI, USA, 20077). Secondary antibodies were made in donkey or goat and conjugated with the following: A405 (anti-mouse, 1:250; Jackson Immunoresearch, Ely, UK, 715-475-151), A488 (anti-chicken, 1:500; Jackson Immunoresearch, Ely, UK, 703-545-155), A488 (anti-goat, 1:200; Abcam, Cambridge, UK, ab150129), A488 (anti-mouse, 1:200; Thermo Fisher Scientific, Waltham, MA, USA, A21202), A568 (anti-rabbit, 1:500; Thermo Fisher Scientific, Waltham, MA, USA, A11011), A647 (anti-rabbit, 1:500; Thermo Fisher Scientific, Waltham, MA, USA, A31573), A647 (anti-mouse, 1:500; Thermo Fisher Scientific, Waltham, MA, USA, A31571), Cy3 (anti-chicken, 1:500; Dianova, Hamburg, Germany, 703-165-155), Cy3 (anti-rabbit, 1:500; Dianova, Hamburg, Germany, 711-165-152), Cy5 (anti-goat, 1:500; Dianova, Hamburg, Germany, 705-175-147), Cy5 (anti-guinea pig, 1:500; Jackson Immunoresearch, Ely, UK, 706-175-148), and A647 (anti-rat, 1:500; Abcam, Cambridge, UK, ab150155).

### BrdU experiments

BrdU (Sigma-Aldrich, Merck, Germany, #B5002) was administered during the retrovirus delivery and on the following day by intraperitoneal injection at a dose of 50 mg/kg (in 0.9% NaCl and 0.1 M NaOH). Analysis was performed at 4 weeks after injection. The following modifications were made to the immunohistochemistry protocol for BrdU staining: Sections were pretreated with 2 N HCl and 0.5% Triton X-100 for 30 min at 37°C followed by neutralization with sodium-tetraborate (Na_2_B_4_O_7_, 0.1 M, pH 8.6) for 30 min at RT before incubation with blocking solution consisting of 3% bovine serum albumin (BSA; Sigma-Aldrich, Merck, Germany, #A9418) and 0.3% Triton X-100 in 1× phosphate-buffered saline (PBS). This blocking solution was also used for dilution of the primary and secondary antibodies: rat anti-BrdU (1:200; Serotec, OBT0030) and donkey anti-rat 647 (1:500; Jackson Immunoresearch, Ely, UK, 712-606-150).

### Single-molecule fluorescence in situ hybridization

smFISH with RNAscope technology was performed using the Multiplex Fluorescent V2 kit (Advanced Cell Diagnostics, ACD) according to the manufacturer’s instructions. All solutions were prepared in ribonuclease-free diethyl pyrocarbonate–treated water (Sigma-Aldrich, Merck, Germany, D5758). Hybridizations toward targets were performed with probes *Pvalb*-C1 (#421931), *Sst*-C3 (#404631), *Htr3A*-C2 (#411141), and *Kcnc1*-C1 (#564521), and control hybridizations were performed with a positive control probe (PN320881) and a negative control probe (PN320871). Sections were placed in 0.05 M TBS and mounted in PB 0.1 M on SuperFrost slides (J1800AMNZ, Menzel). Sections were left to dry overnight at RT and then at 40°C for 40 min in a HybEZII oven (ACD). They were then rinsed with distilled water (dH_2_O) and dehydrated with 50, 70, and 100% EtOH, 5 min each. Then, sections were treated with hydrogen peroxide (Multiplex Fluorescent V2 kit, ACD) for 10 min at RT, washed three times for 3 to 5 min with dH_2_O followed by three times for 3 to 5 min in 1× TBS before incubation with antigen retrieval solution (Multiplex Fluorescent V2 kit, ACD) for 10 to 15 min at 90°C. Sections were rinsed with dH_2_O, dipped in 100% EtOH, and air dried for approximately 5 min. They were then incubated with Protease III (Multiplex Fluorescent V2 kit, ACD) for 10 to 15 min at 40°C in a HybEZII oven. The sections were washed three times for 3 to 5 min in dH_2_O, incubated for 2 hours with the probes at 40°C in a HybEZII oven, then washed three times for 3 to 5 min in wash buffer (Multiplex Fluorescent V2 kit, ACD), and kept overnight in 5× saline sodium citrate (prepared according to ACD’s user manual). On the next day, the sections were successively incubated with AMP1 (Multiplex Fluorescent V2 kit, ACD) for 30 min at 40°C in a HybEZII oven, AMP2 (Multiplex Fluorescent V2 kit, ACD) for 30 min at 40°C in a HybEZII oven, and AMP3 (Multiplex Fluorescent V2 kit, ACD) for 15 min at 40°C in a HybEZII oven, with intermediate washes of 3× 3 to 5 min in wash buffer (ACD). The signal was then developed by incubation with horseradish peroxidase (HRP)–C1 or HRP-C3 (adapted to the channel of the probe, Multiplex Fluorescent V2 kit, ACD) for 15 min at 40°C in a HybEZII oven, followed by Opal dye 690 diluted in Tyramide signal amplification (TSA) buffer (1:1000; #322809, ACD) for 30 min at 40°C in a HybEZII oven and then HRP blocker (Multiplex Fluorescent V2 kit, ACD) for 15 min at 40°C in a HybEZII oven, with intermediate washes of 3× 3 to 5 min in wash buffer (ACD). Then, the sections were incubated with 5 μM DAPI in 1× TBS for 7 min at RT and transferred into 0.1 M TBS for further processing for immunostaining. After permeabilization with PBS with 0.25% Triton X-100 for 20 min at RT, sections were incubated with blocking solution consisting of 0.3% Triton X-100, 3% BSA (Scientific Laboratory Supplies, #A2153), and 10% donkey or goat serum (or 5% donkey and 5% goat serum) in 1× PBS for 2 hours at RT and then incubated with primary antibodies prepared in 0.3% Triton, 3% BSA, 5% serum in 1× PBS overnight + 24 hours at 4°C. They were washed three times in 1× PBS and incubated with secondary antibodies for 2 hours. Last, they were transferred to 0.1 M PB and mounted with ProLong Gold (Thermo Fisher Scientific, Waltham, MA, USA, P36930). The following primary antibodies were used: chicken anti-GFP (1:200) and rabbit anti-RFP (1:100). Secondary antibodies were made in donkey or goat and conjugated with: A488 (anti-chicken, 1:200) and A568 (anti-rabbit, 1:250).

### Confocal microscopy and quantifications

Immunostainings and RNAscope signals were imaged with a TCS SP5 (Leica Microsystems, Wetzlar, Germany) confocal microscope (Institute of Molecular Biology, Mainz, Germany) equipped with four photomultiplier tubes, four lasers (405 Diode, Argon, HeNe 543, and HeNe 633), and a fast-resonant scanner using a 20× dry objective [numerical aperture (NA) 0.7] or a 40× oil objective (NA 1.3), or with a Zeiss LSM 800 confocal microscope (Carl Zeiss Microscopy, Jena, Germany) equipped with four solid-state lasers (405, 488, 561, and 633 nm) at 20× (NA 0.8) or 40× (NA 1.3) objectives (Centre for Developmental Neurobiology, King’s College London), or with a Zeiss Axio Imager.M2 fluorescent microscope equipped with an ApoTome (Carl Zeiss Microscopy, Jena, Germany) at a 20× dry objective (NA 0.7) or a 63× oil objective (NA 1.25). Serial Z-stacks spaced at 0.3- to 2.13-mm distance were acquired to image the whole thickness of the brain sections. Images were captured with Leica Application Suite or Zeiss ZEN software. For the figures, maximum intensity projections from the Z-stacks were generated using the function provided by the software. Cell quantifications were performed using ZEN software or ImageJ 1.51v software (National Institute of Health, USA). Cell counts were done by navigating through the Z-stacks of images, allowing the accurate visualization of each transduced cell. For fate-mapping experiments, we counted the number of (i) RFP/EGFP/Dcx or NeuN triple-positive cells (i.e., fate-mapped iNs), (ii) RFP/Dcx or RFP/NeuN double-positive iNs, (iii) RFP/EGFP double-positive transduced cells, and (iv) RFP only-positive transduced cells. Cells in each group are expressed as a percentage of the total number of reporter- or double reporter–positive transduced cells. Results are expressed as means ± SD. Cells were quantified from at least three to five sections from three independent mice (minimum nine sections in total).

For the RNAscope signal, the DsRed reporter signal from transduced cells or the DAPI^+^ nuclei from endogenous neurons was used to draw a region of interest (ROI). The number of mRNA transcripts was manually counted by navigating through the Z-stacks of confocal images obtained with a 40× (NA 1.3) objective and spaced at 0.5- to 0.75-μm distance. The number of mRNA transcripts was expressed as a total number of dots per DsRed^+^ cell or endogenous neuron. For each experiment, quantification was done in cells from three independent mice.

To determine Ascl1 intensity levels in cotransduced cells, the contour of Ascl1 signal was used to draw a ROI, and the “RawIntDen” value (the sum of the values of the pixels in the ROI) was calculated using ImageJ 1.51v software (National Institute of Health, USA). The Z-stack with the highest value from each cell was included for comparison. All images included in the analysis were obtained with the same laser parameters for the Ascl1 channel (Cy5 channel).

### Electrophysiological recordings

#### Slice preparation

Mice anesthetized with isoflurane (Forane, Abbvie, IL, USA) were decapitated and brains were removed into chilled artificial cerebrospinal fluid (ACSF) solution containing the following (in mM): NaCl, 85; sucrose, 73; KCl, 2.5; NaHCO_3_, 25; CaCl_2_, 0.5; MgCl_2_, 7; NaH_2_PO_4_, 1.25; and glucose, 10 saturated with 5% CO_2_ and 95% O_2_ (pH 7.4). Coronal 300-μm-thick cortical slices were cut using a vibratome (VT1200 S, Leica, Wetzlar, Germany) and transferred to standard ACSF (34°C, 10 to 15 min) containing the following (in mM): NaCl, 125; KCl, 2.5; NaHCO_3_, 25; CaCl_2_, 2; MgCl_2_, 1; NaH_2_PO_4_, 1.25; and glucose, 12 (pH 7.4). Subsequently, the slices were stored in standard ACSF at RT (21 ± 2°C) for at least 1 hour before experiments started. Individual slices were placed in a recording chamber, superfused (1 to 2 ml min^−1^, standard ACSF), and mounted on an upright microscope (Axio Imager 2, Zeiss, Jena, Germany or Slice Scope Pro 6000 System, Scientifica, Uckfield, UK). Cells were visualized with oblique contrast or Dodt gradient contrast optics using a 40× (NA 0.70) water immersion objective and with a Hamamatsu Orca-Flash 4.0 camera (Hamamatsu, Japan). For the identification of the retrovirally transduced cells, GFP and DsRed fluorescence was revealed by light-emitting diode excitation (CoolLED pE-100, Andover, UK) with appropriate excitation and emission filters.

#### Electrophysiology

Patch-clamp whole-cell recordings were performed at 30 ± 2°C with an in-line heater (Scientifica, Uckfield, UK). Recording pipettes (10 to 15 megohms) were pulled from borosilicate capillary glass (BF150-86-10, Sutter Instruments, Novato, CA, USA or 1B150F-4, World Precision Instruments, Sarasota, FL, USA) in a horizontal pipette puller (P-1000 Micropipette puller, Sutter Instruments, Novato, CA, USA) and were filled with the following (in mM): K-gluconate, 125; NaCl, 5; Na_2_-ATP, 2; MgCl_2_, 2; EGTA, 1; Hepes, 10; and biocytin, 10 to allow a posterior morphological analysis (pH 7.4), osmolarity, 280 mosmol. Voltage- and current-clamp recordings were obtained using Axopatch 200B or MultiClamp 700B amplifier (Molecular Devices, San Jose, USA), digitized (Digidata 1440A or 1550B, Molecular Devices, San Jose, CA, USA), and acquired at 20 kHz onto a personal computer using the pClamp 10 software (Molecular Devices, San Jose, CA, USA) which was also used for further analysis. Cells were selected for recording based on their putatively neuronal morphology (e.g., round soma with neurite-like processes), while cells with astrocytic morphology were discarded as candidates for recording. Criteria to include cells in the analysis were the following: (i) visual confirmation of GFP or DsRed in the pipette tip, (ii) attachment of the labeled soma to the pipette when suction was performed, (iii) seal resistances between 4 and 18 gigohms, (iv) initial series resistance less than 40 megohms and did not change by more than 20% throughout the recording period. Whole-cell capacitance and series resistances were not compensated. In current-clamp recordings, the membrane potential was kept at −70 mV or adjusted to different values, if necessary, by passing a holding current. Passive and active membrane properties were recorded by applying a series of hyperpolarizing and depolarizing current steps (2- or 10-pA steps, 500 ms). The IFF was calculated as the inverse value of the inter-spike interval, which was determined by the time difference between adjacent action potential peaks. Maximum firing frequency was calculated by dividing the number of spikes by the step duration. The RMP was assessed immediately after break-in by reading the voltage value in the absence of current injection (*I* = 0 configuration). Input resistance (*R*_in_) was calculated from the peak of the voltage response to a −20-pA, 500-ms current step according to Ohm’s law (*V*_hold_ = −70 mV). The membrane capacitance (*C*_m_) was obtained with the following equation: membrane time constant (τ_m_) = *R*_in_ · *C*_m_. τ_m_ was derived from a single exponential fitted to voltage response to −20-pA, 500-ms current step. The action potential properties were analyzed on the first spike observed at the rheobase. Spike amplitude was measured from threshold to positive peak and AHP amplitude, from threshold to negative peak during repolarization. Spike width was measured at the half amplitude of the spike. Frequency and amplitude of the spontaneous excitatory postsynaptic currents were recorded during 60 s in mode gap-free at *V*_hold_ = −70 mV and the analysis was done using Clampfit template search (Molecular Devices, San Jose, CA, USA). No correction was made for the junction potential between the pipette and the ACSF.

In some experiments, TTX (1 μM, Tocris, Bio-Techne, Minneapolis, MN, USA) to block Na channels or CNQX (10 μM, Tocris, Minneapolis, MN, USA) to block AMPA/kainate receptors was introduced to the standard ACSF.

### Morphological identification of the recorded cells

Retroviral transduced cells expressing DsRed or GFP were first imaged in acute slices. During whole-cell patch-clamp recordings, cells were filled with biocytin included in the pipette. Then, slices were fixed by immersion in 4% PFA for 24 hours. Following TBS rinsing, the slices were blocked with 0.5% BSA in TBS (1 hour) and then incubated in TBS containing 0.3% Triton X-100 with the streptavidin-fluorophore complex, 1:400, for 2 hours, and subsequently mounted for confocal imaging.

### Statistical analysis

Statistical analysis was performed using OriginLab (Northampton, MA, USA), GraphPad Prism 5 (GraphPad, San Diego, CA, USA), or SPSS Statistics V5 (IBM). Data are represented as means ± SD or with box and whisker plots, which give the median, 25th and 75th percentiles, and range. The normality of distribution was assessed using the Shapiro-Wilk test. The significance of the differences between groups was analyzed by independent *t* test (for samples with normal distribution) or using the Mann-Whitney *U* test (for samples without normal distribution). The statistical test used is described in the figure legend. The number of independent experiments (*n*), the number of cells analyzed, and the number of cells recorded for electrophysiology are reported in the figure legends.
