# Supplementary material for: Reprogramming astroglia into neurons with hallmarks of fast-spiking parvalbumin-positive interneurons by phospho-site–deficient Ascl1
Source: Sci Adv. 2024 Oct 25;10(43):eadl5935. doi: 10.1126/sciadv.adl5935 (PMC11506222; doi:10.1126/sciadv.adl5935)
Supplement: Supplementary file 1 — Figs. S1 to S12 [file sciadv.adl5935_sm.pdf]

Science Advances
AAAS

Supplementary Materials for

Reprogramming astroglia into neurons with hallmarks of fast-spiking parvalbumin-positive interneurons by phospho-site–deficient Ascl1

Nicolás Marichal *et al.*

Corresponding author: Benedikt Berninger, benedikt.berninger@kcl.ac.uk

Sci. Adv. **10**, eadl5935 (2024)
DOI: 10.1126/sciadv.adl5935

This PDF file includes:

Figs. S1 to S12

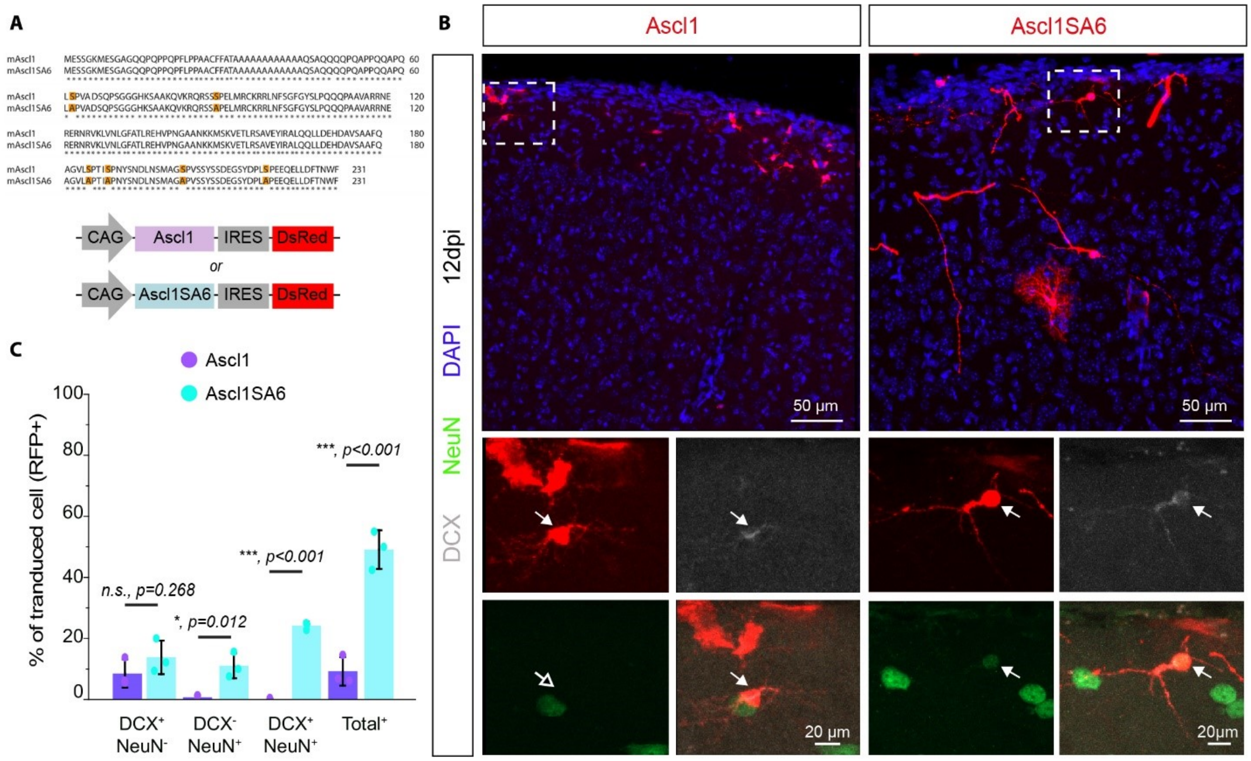

Fig. S1. *Ascl1SA6* converts cortical postnatal glia into iNs more efficiently than wt *Ascl1* (Related to Fig. 1). (A) Scheme showing the six serine residues mutated by alanine in the sequence of mouse *Ascl1* (mAscl1) to generate the mutant *Ascl1SA6* and final retroviral constructs. (B) *Ascl1* (left) or *Ascl1SA6* (right)-transduced cells at the cortical site of injection at 12 dpi. High-magnification images from insets (white boxes) depicting DCX and NeuN expression in transduced cells. Notice the maintenance of glial morphology in *Ascl1*-transduced cells and the acquisition of neuronal morphology in Ascl1SA6 iNs. Empty arrows indicate marker-negative cells. (C) Proportion of transduced cells expressing DCX, NeuN or both neuronal markers at 12 dpi. 8.5 ± 4.6% DCX only, 0.7 ± 0.6% NeuN only, 0.0 ± 0.0% DCX and NeuN, 341 cells, n = 3 mice for *Ascl1*; 13.8 ± 5.5% DCX only, 11.1 ± 4.1% NeuN only, 24.2 ± 1.3% DCX and NeuN, 118 cells, n = 3 animals for *Ascl1SA6*. Data shown as mean ± SD. Two-tailed Student's unpaired t-test in (C).

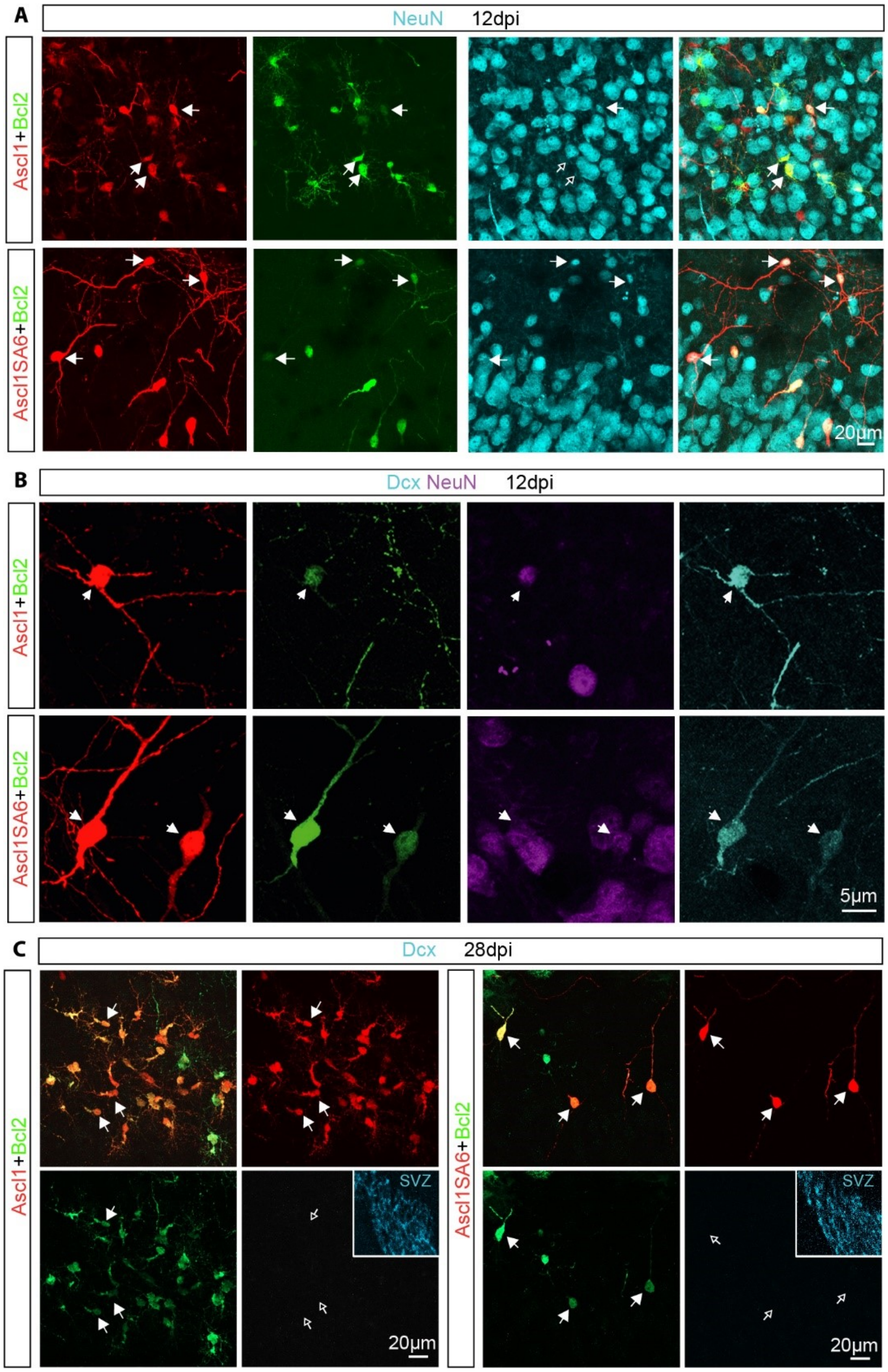

Fig. S2. Highly efficient conversion of glia into neurons by *Ascl1SA6* and *Bcl2* (Related to Fig.1). (A) High-magnification images depicting NeuN expression in co-transduced cells. Empty arrows indicate marker-negative cells at 12 dpi. (B) High-magnification images depicting DCX and NeuN expression in co-transduced cells at 12dpi. (C) At 28 dpi, none of the cells co-transduced with *Ascl1/Bcl2* or *Ascl1SA6/Bcl2* iNs express the neuronal marker Dcx (empty arrows). Insets show positive Dcx staining in the subventricular zone (SVZ) as a control.

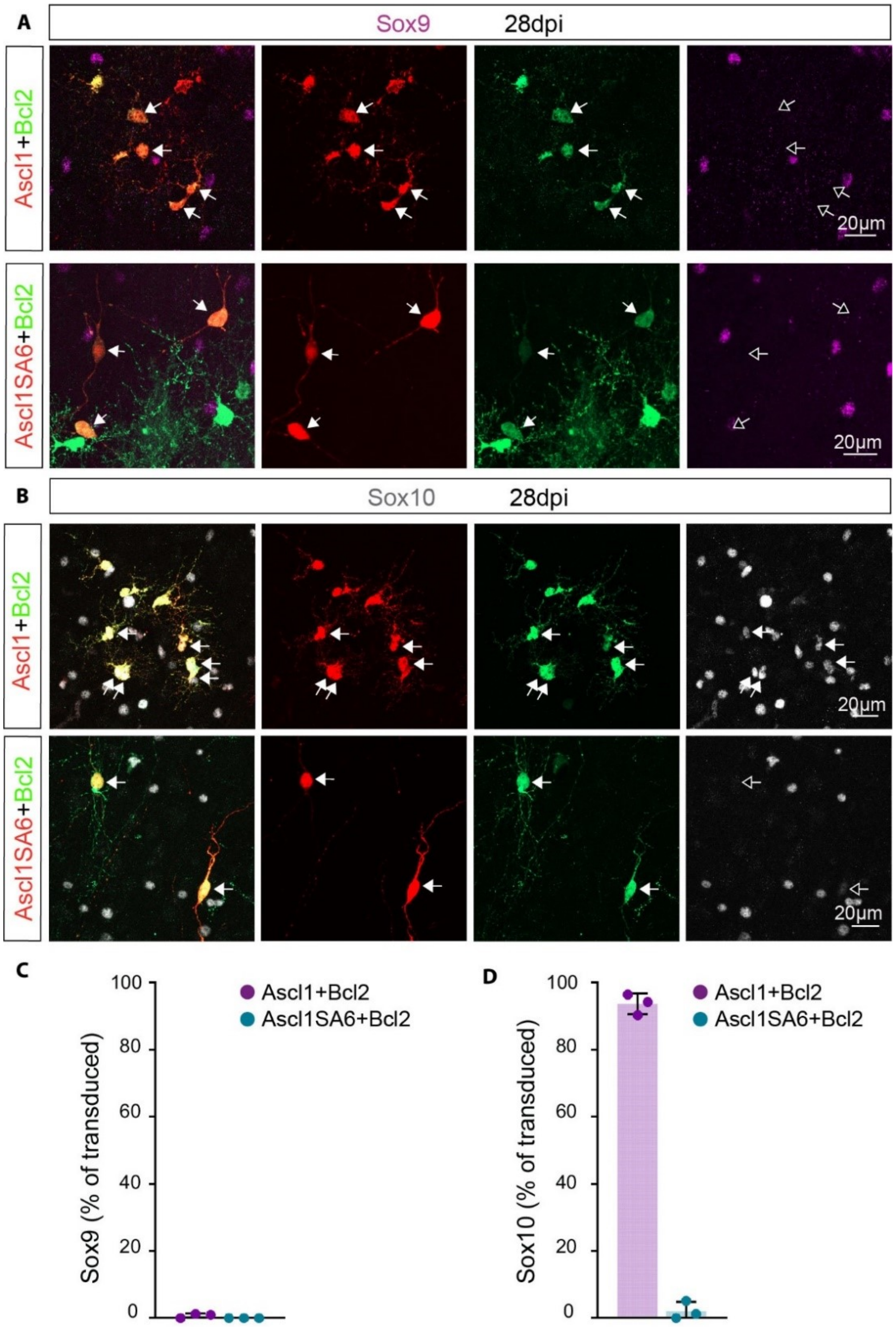

Fig. S3. Loss of glial identity by cells co-transduced cells with *Ascl1SA6* and *Bcl2* (Related to Fig. 1). (A) High-magnification images depicting lack of Sox9 expression in co-transduced cells with *Ascl1/Bcl2* (upper panel) or *Ascl1SA6/Bcl2* (lower panel) at 28 dpi. Empty arrows indicate marker-negative cells. (B) The vast majority of *Ascl1/Bcl2*-transduced cells (upper panel) express Sox10, while most Ascl1SA6/Bcl2 iNs (left panel) lack Sox10 expression at 28 dpi. Empty arrows indicate marker-negative cells. (C) Proportion of double-transduced cells expressing Sox9 at 28 dpi. 0.7 ± 0.6%, 527 cells, n = 3 mice for *Ascl1/Bcl2*; 0.0 ± 0.0%, 342 cells, n = 3 mice for *Ascl1SA6/Bcl2*. (D) Proportion of double-transduced cells expressing Sox10 at 28 dpi. 93.6 ± 3.1%, 374 cells, n = 3 mice for *Ascl1/Bcl2*; 2.1 ± 2.7%, 240 cells, n = 3 mice for *Ascl1SA6/Bcl2*. Data shown as mean ± SD.

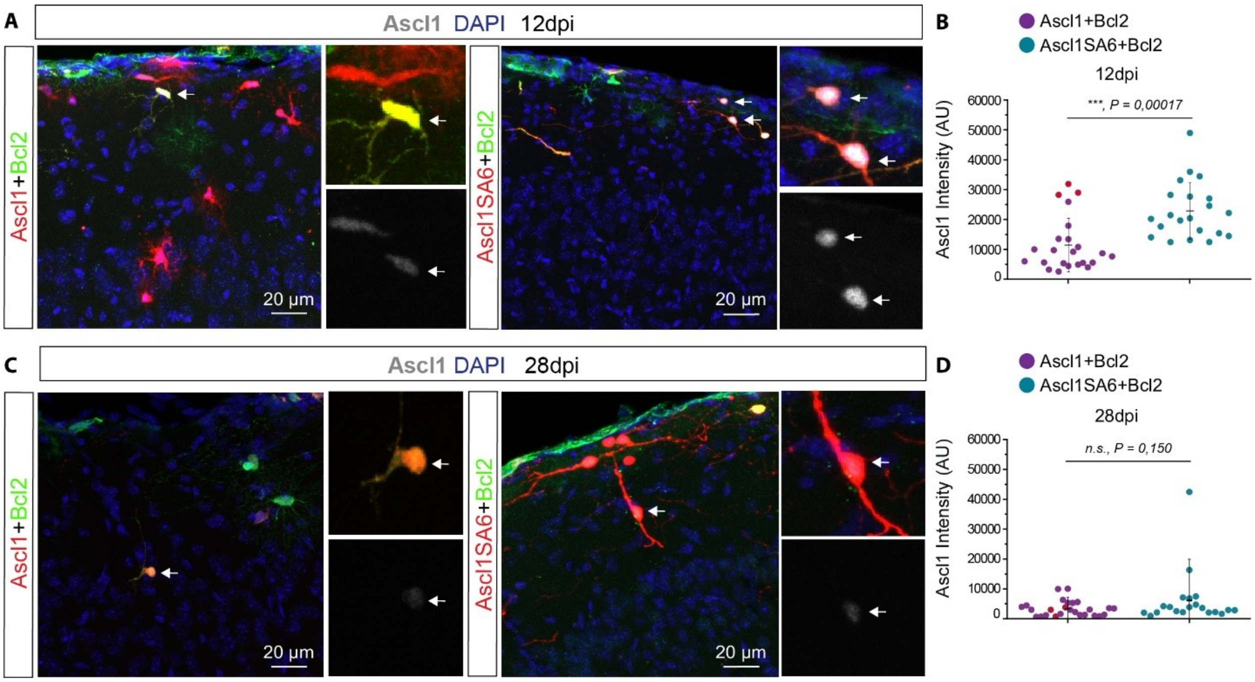

Fig. S4. Levels of expression of Ascl1 protein. (Related to Figure 1). (A) Ascl1 expression in *Ascl1/Bcl2* or *Ascl1SA6/Bcl2* co-transduced cells at 12 dpi. (B) Quantification of Ascl1 immunofluorescence intensity at 12 dpi. Each dot represents one cell. Data shown as mean ± SD. 23 cells, n = 3 mice for *Ascl1/Bcl2*; 21 cells, n = 3 mice for *Ascl1SA6/Bcl2*. Among Ascl1/Bcl2 cells, those represented by a red dot exhibited neuronal morphology. (C) Ascl1 expression in *Ascl1/Bcl2* or *Ascl1SA6/Bcl2* co-transduced cells at 28 dpi. (D) Quantification of Ascl1 immunofluorescence intensity at 28 dpi. Each dot represents one cell. 26 cells, n = 3 mice for *Ascl1/Bcl2* group; 20 cells, n = 3 mice for *Ascl1SA6/Bcl2* group. Among Ascl1/Bcl2 cells, those represented by a red dot exhibited neuronal morphology. Two-tailed Student's unpaired t-test for (B) and (D).

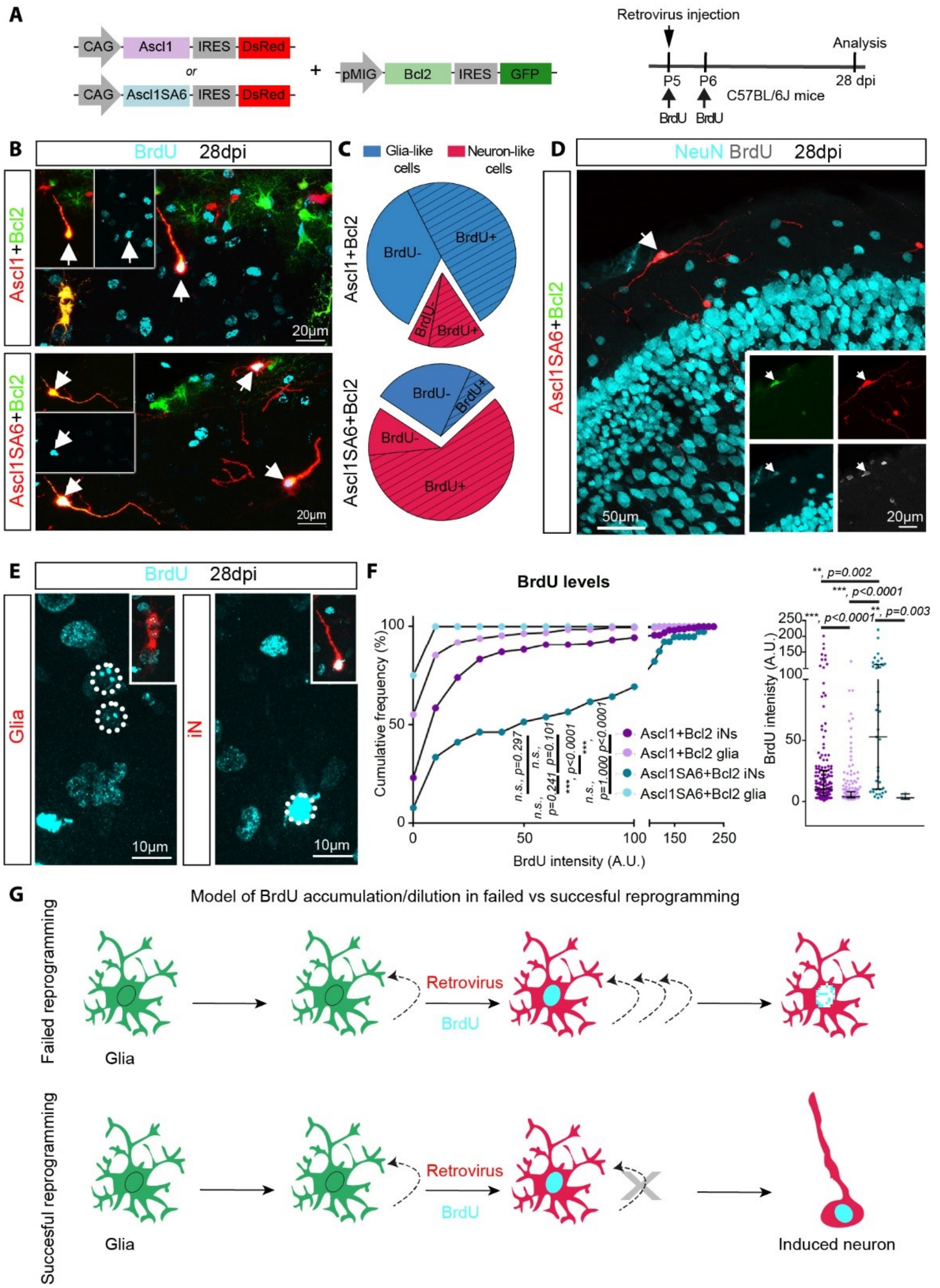

Fig. S5. iNs incorporate BrdU administered at the time of retrovirus injection (Related to Fig. 1). (A) Experimental design. (B) BrdU labeling of co-transduced cells that had acquired neuron-like morphology (arrows) at 28 dpi. For better visualization of BrdU labeling, inset shows channels separation of DsRed/GFP and BrdU signals. (C) Pie charts showing the proportion of glia-like or neuron-like co-transduced cells that were BrdU-positive (+) at 28 dpi. Notice that the vast majority of co-transduced cells with neuronal morphology were BrdU+ (red-slashed section). 1217 cells, 17 ± 5.3% neuron-like cells, from which 72.9 ± 1.7 % were BrdU+, n = 3 mice for *Ascl1/Bcl2*; 65 cells, 71 ± 12.5% neuron-like cells, from which 85.8 ± 5.6 % were BrdU+, n = 3 mice for *Ascl1SA6/Bcl2*. Data shown as mean ± SD. (D) BrdU labeling of NeuN+ iN (arrow). Inset shows channels separation of the signals (E) BrdU-signal levels on transduced glia and iNs. Inset shows channels separation of DsRed/GFP and BrdU signals. (F) Graph

showing the cumulative frequency of BrdU-signal intensity (left graph) and individual BrdU-signal intensity values (right graph) in transduced cells. Data shown as median with interquartile range. 400 cells analyzed (161 Ascl1-BrdU+ iNs, 196 Ascl1-BrdU+ glia, 39 Ascl1SA6-BrdU+ iNs, 4 Ascl1SA6-BrdU+ glia). (G) Scheme depicting a model of BrdU accumulation/dilution in failed vs successful reprogramming. Kruskal-Wallis for comparison of intensity cumulative frequency distribution in (F) left graph and median test with Bonferroni correction for comparison of median intensity values in (F) right graph.

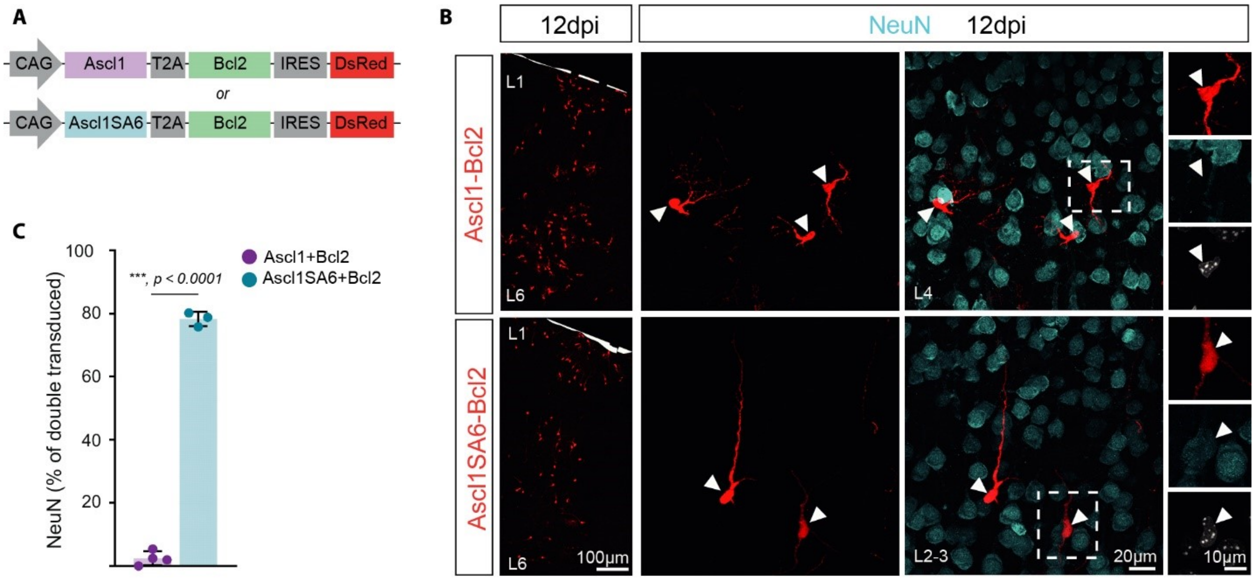

Fig. S6. A single retroviral vector encoding for *Ascl1SA6-Bcl2* also induces high efficiency of neuronal reprogramming (Related to Fig. 2) (A) Scheme showing the retroviral constructs encoding for both *Ascl1* and *Bcl2* (*Ascl1-Bcl2*) or *Ascl1SA6* and *Bcl2* (*Ascl1SA6-Bcl2*) in a single construct. (B) NeuN expression in *Ascl1-Bcl2* or *Ascl1SA6-Bcl2* transduced cells at 12 dpi. The left panels show low-magnification images of the transduced cells at the cortical site of injection. Central panels show high magnification of transduced cells. Cells from insets (white boxes) are shown on the right panels. (C) Quantification of the percentage of transduced cells expressing NeuN at 12 dpi. 7.0 ± 4.4%, 1002 cells, n = 4 mice for *Ascl1-Bcl2*; 78.3 ± 2.3%, 1224 cells, n = 3 mice for *Ascl1SA6-Bcl2.* Data shown as mean ± SD. Two-tailed Student's unpaired t-test in (C).

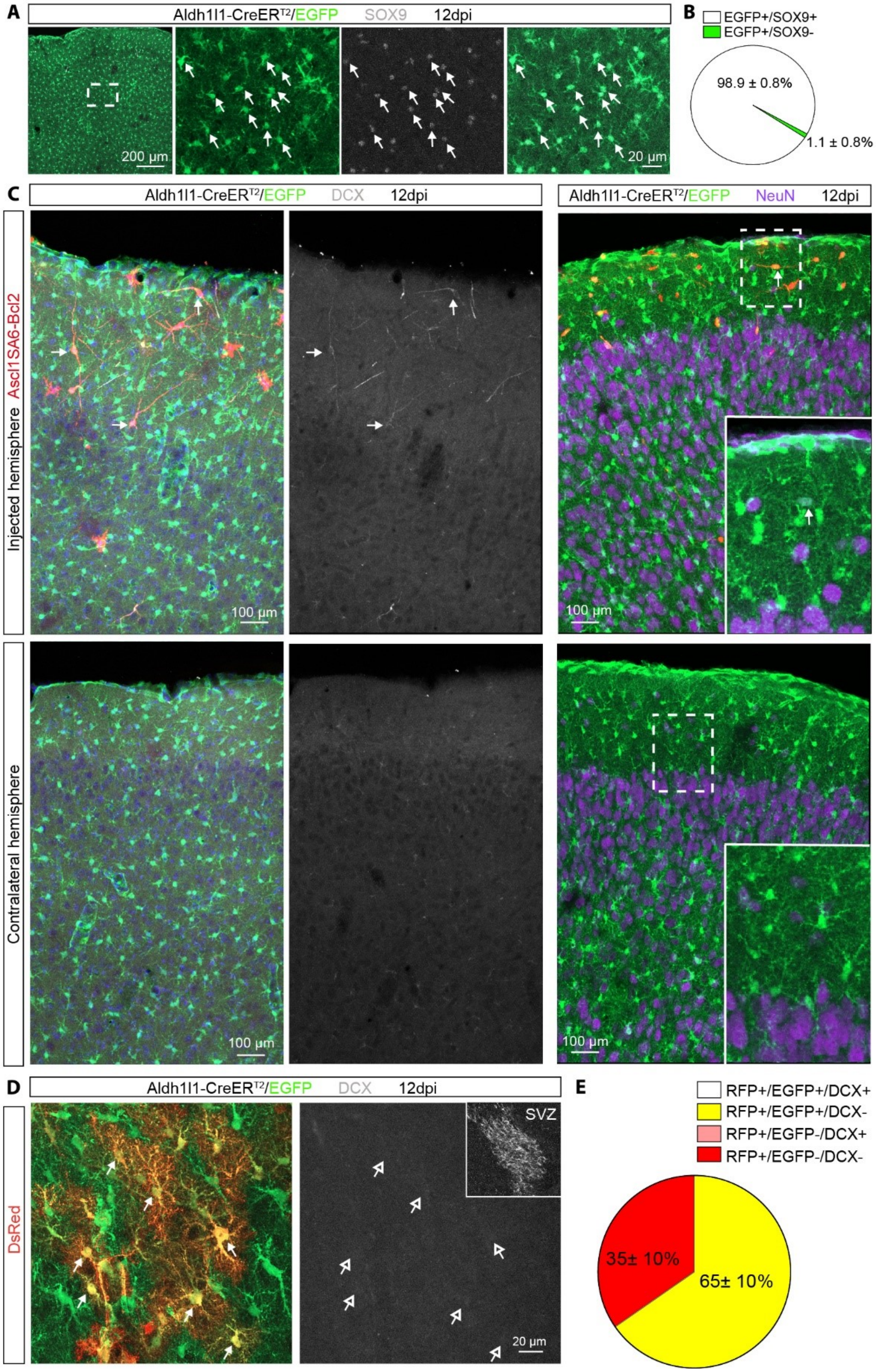

Fig. S7. Specific labelling of astroglia in the *Aldh1l1-CreERT2/RCE:loxP* mouse cortex (Related to Fig. 2). (A) The vast majority of EGFP+ cells co-express Sox9 in the cortex of *Aldh1l1-CreERT2/RCE:loxP* mice at 12 dpi. (B) Pie chart showing the relative number of Sox9+ cells co-expressing EGFP. 3659 cells, n = 3 mice. (C) Low-magnification

images showing that all EGFP+/Dcx+ or EGFP+/NeuN+ cells found in the injected hemisphere co-expressed DsRed (upper panels), whereas none EGFP+/Dcx+ or EGFP+/NeuN+ cells were observed in the contralateral hemisphere (lower panels). (D) Control-transduced cells lacking Dcx expression in *Aldh1l1-CreERT2/RCE:loxP* mice at 12 dpi. Inset shows Dcx expression in the subventricular zone (SVZ) as a positive control for the Dcx staining. (E) Pie chart showing the relative number of control-transduced cells (DsRed+) expressing Dcx, EGFP or DsRed only in *Aldh1l1-CreERT2/RCE:loxP* mice at 12dpi. 330 cells, n = 3 mice. Data shown as mean ± SD.

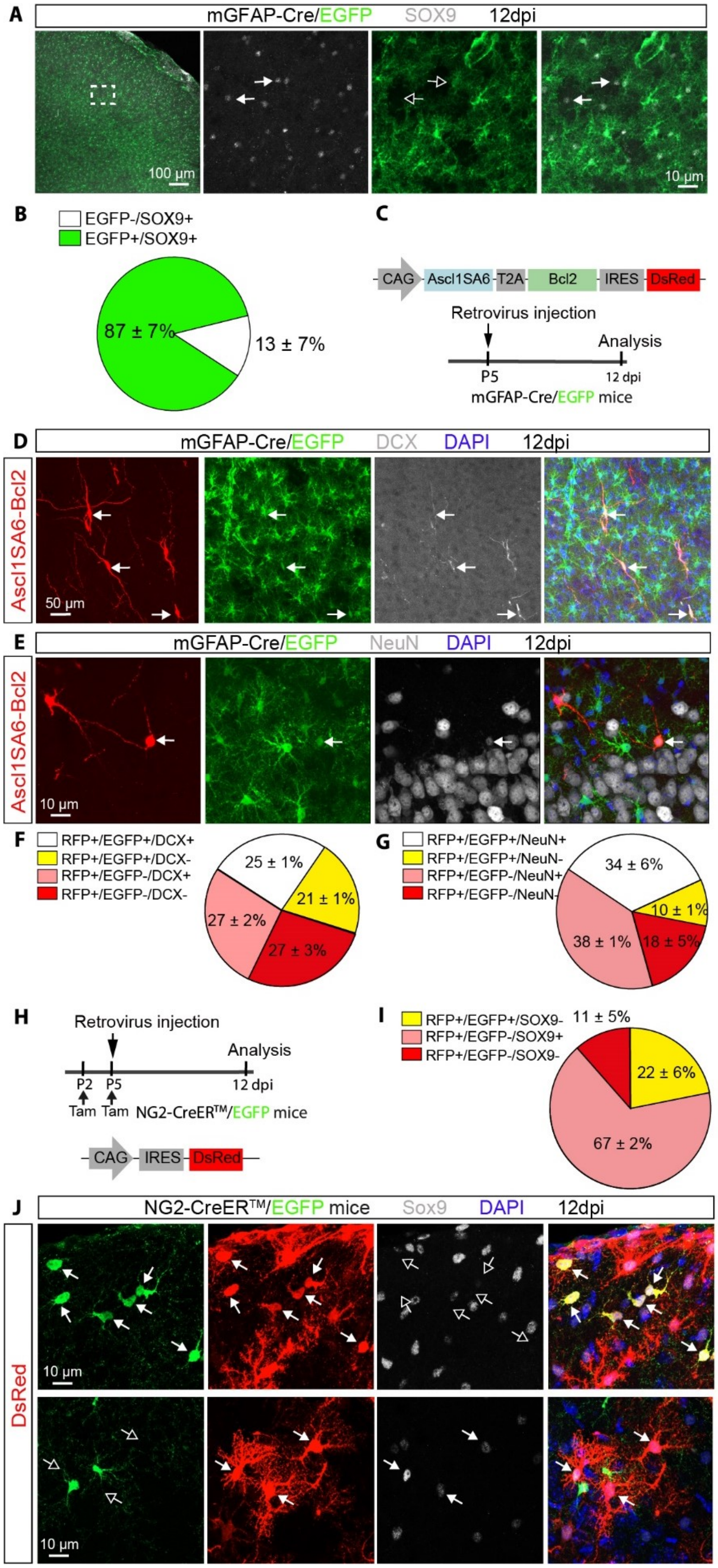
A
mGFAP-Cre/EGFP SOX9 12dpi
100 µm
10 µm
B
EGFP-/SOX9+
EGFP+/SOX9+
87 ± 7%
13 ± 7%
C
CAG
Ascl1SA6
T2A
Bcl2
IRES
DsRed
Retrovirus injection
Analysis
P5
12 dpi
mGFAP-Cre/EGFP mice
D
mGFAP-Cre/EGFP DCX DAPI 12dpi
Ascl1SA6-Bcl2
50 µm
E
mGFAP-Cre/EGFP NeuN DAPI 12dpi
Ascl1SA6-Bcl2
10 µm
F
RFP+/EGFP+/DCX+
RFP+/EGFP+/DCX-
RFP+/EGFP-/DCX+
RFP+/EGFP-/DCX-
25 ± 1%
21 ± 1%
27 ± 2%
27 ± 3%
G
RFP+/EGFP+/NeuN+
RFP+/EGFP+/NeuN-
RFP+/EGFP-/NeuN+
RFP+/EGFP-/NeuN-
34 ± 6%
10 ± 1%
38 ± 1%
18 ± 5%
H
Retrovirus injection
Analysis
P2
P5
12 dpi
Tam
Tam
NG2-CreER[TM]/EGFP mice
CAG
IRES
DsRed
I
RFP+/EGFP+/SOX9-
RFP+/EGFP-/SOX9+
RFP+/EGFP-/SOX9-
11 ± 5%
22 ± 6%
67 ± 2%
J
NG2-CreER[TM]/EGFP mice Sox9 DAPI 12dpi
DsRed
10 µm
10 µm

Fig. S8. Astroglial origin of the vast majority of Ascl1SA6 and Bcl2 iNs (Related to Fig. 2). (A) A minor fraction of Sox9+ (arrows) cells do not co-express EGFP (empty arrows) in the cortex of *mGFAP-Cre/RCE:loxP* mice at 12dpi. (B) Pie chart showing the relative number of Sox9+ cells co-expressing EGFP. 3352 cells, n = 3 mice. (C) Experimental design. (D-E) Ascl1SA6-Bcl2-derived iNs expressing Dcx (D) or NeuN (E) and EGFP (arrows) in the *mGFAP-Cre/RCE:loxP* mouse line at 12 dpi, demonstrating their astroglial origin. (F-G) Pie charts showing the relative number of transduced cells (DsRed+) co-expressing EGFP and/or Dcx (F), and/or NeuN (G), or DsRed only in *mGFAP-Cre/RCE:loxP* mice at 12dpi. 503 cells, n = 3 mice for Dcx analysis; 632 cells, n = 3 mice for NeuN analysis. Data shown as mean ± SD. (H) Experimental design. (I) Control-transduced cells co-expressing EGFP and lacking Sox9 expression (upper raw) or vice versa (lower raw) at 12 dpi. Empty arrows indicate marker-negative cells. (J) Pie chart showing the relative number of transduced cells (DsRed+) expressing Sox9, GFP or DsRed only in *NG2CreERTM/EGFP* mice at 12dpi. 1374 cells, n = 3 mice. Data shown as mean ± SD.

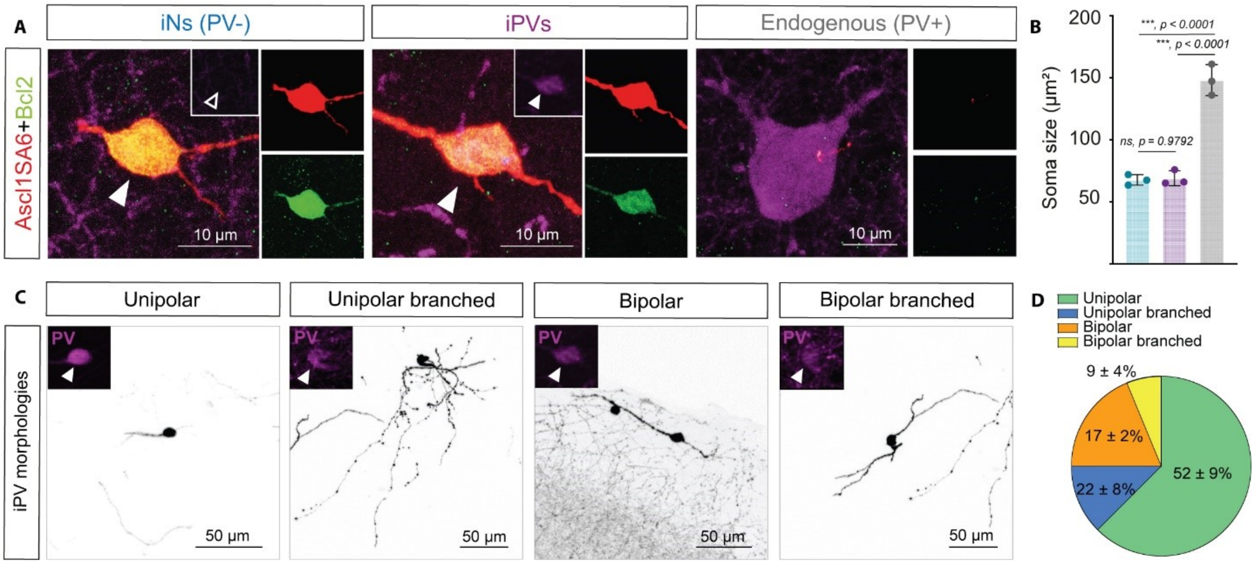

Fig S9. Morphological features of PV-expressing iNs (Related to Fig. 3). (A) High-magnification images depicting Ascl1SA6/Bcl2 PV-negative iNs, Ascl1SA6/Bcl2 PV-positive iNs and endogenous PV interneurons at 28 dpi. (B) Graph showing the soma area (μm2) of PV-negative iNs, PV-positive iNs and endogenous PV interneurons at 28 dpi. 67.8 ± 4.2 μm2, 139 cells, n = 3 mice for PV-negative iNs; 69.09 ± 6.0 μm2, 42 cells, n = 3 mice for PV-positive iNs; 148.2 ± 12.5 μm2, 75 cells, n = 3 mice for endogenous PV interneurons. (C) High-magnification images depicting various morphologies of PV-positive iNs. Insets show parvalbumin expression in the iNs. (D) Pie chart showing the proportion of PV-positive iNs displaying different morphologies: unipolar, unipolar branched, bipolar or bipolar branched. Data shown as mean ± SD. One-way ANOVA followed by Tukey's multiple comparisons post hoc test in (B).

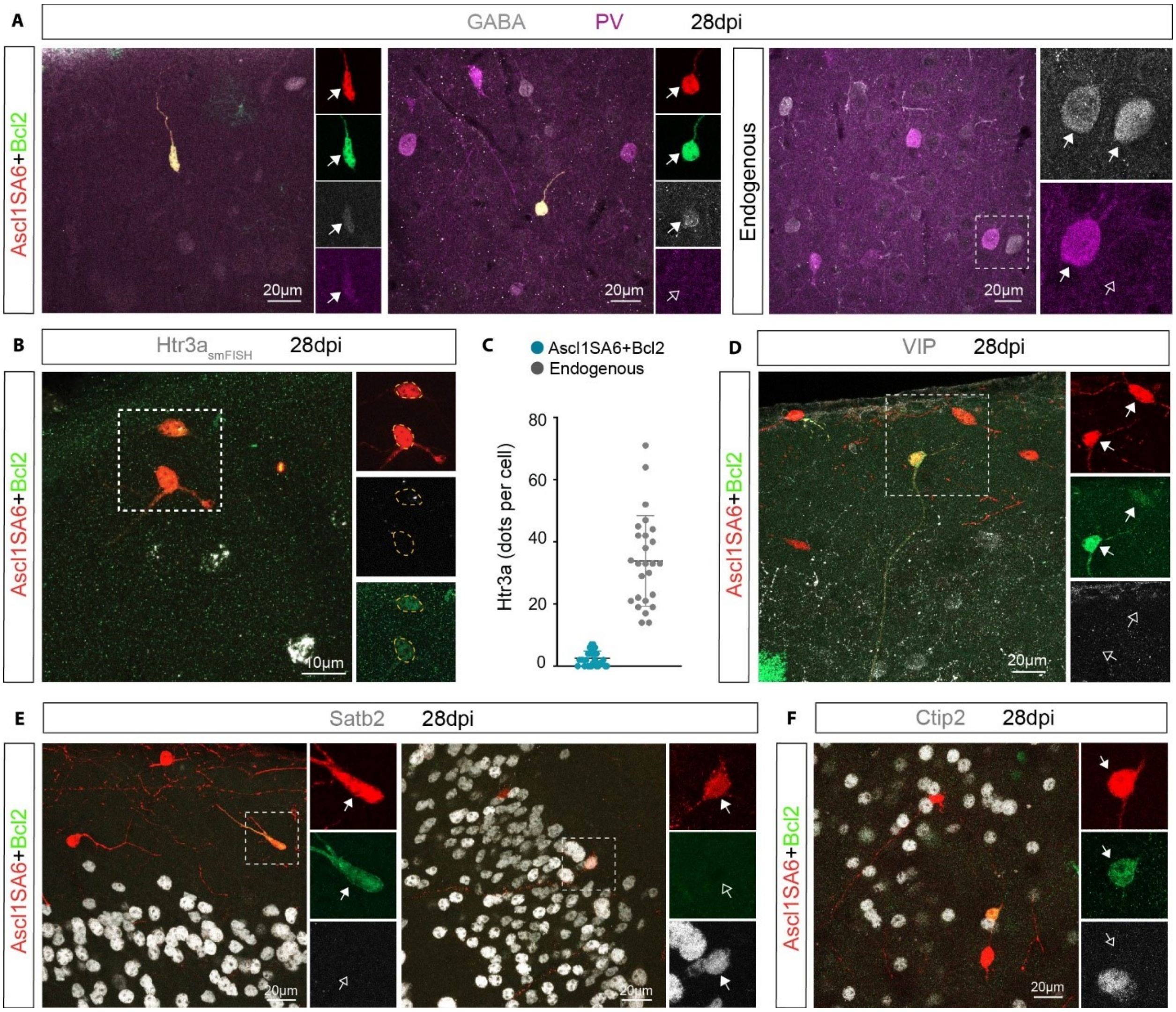

Fig. S10. Forced co-expression of *Ascl1SA6* and *Bcl2* converts postnatal cortical glia into GABA and PV-expressing iNs. (Related to Fig. 3). (A) High magnification images depicting examples of co-expression of GABA and PV or GABA-only in iNs co-transduced with *Ascl1SA6* and *Bcl2* at 28 dpi (left panel). Among the endogenous population of PV-expressing neurons, all co-expressed GABA but only a fraction of GABA-expressing neurons co-expressed PV (right panel). (B) High magnification images depicting very low expression of *Htr3a* mRNA in some Ascl1SA6/Bcl2 iNs at 28 dpi as compared to endogenous neurons in cortex. (C) Quantification of *Htr3a* mRNA transcripts expressed as the total number of dots detected per individual cell in *Ascl1SA6/Bcl2*-transduced cells and endogenous surrounding *Htr3a* interneurons. Each dot represents one cell. 30 cells, n = 2 mice for Ascl1SA6/Bcl2 cells; 26 cells, n = 3 mice for endogenous neurons. (D) *Ascl1SA6/Bcl2*-transduced cells lack VIP expression. Empty arrows highlight lack of VIP expression in iNs (insets). (E) High magnification images depicting lack of Satb2 expression in co-transduced cells with *Ascl1SA6* and *Bcl2* at 28 dpi (left panel). Very rarely cells transduced with *Ascl1SA6*, but not *Bcl2*, expressed Satb2 (right panel). (F) *Ascl1SA6/Bcl2*-transduced cells lack Ctip2 expression. Empty arrows highlight lack of Ctip2 expression in iNs (insets).

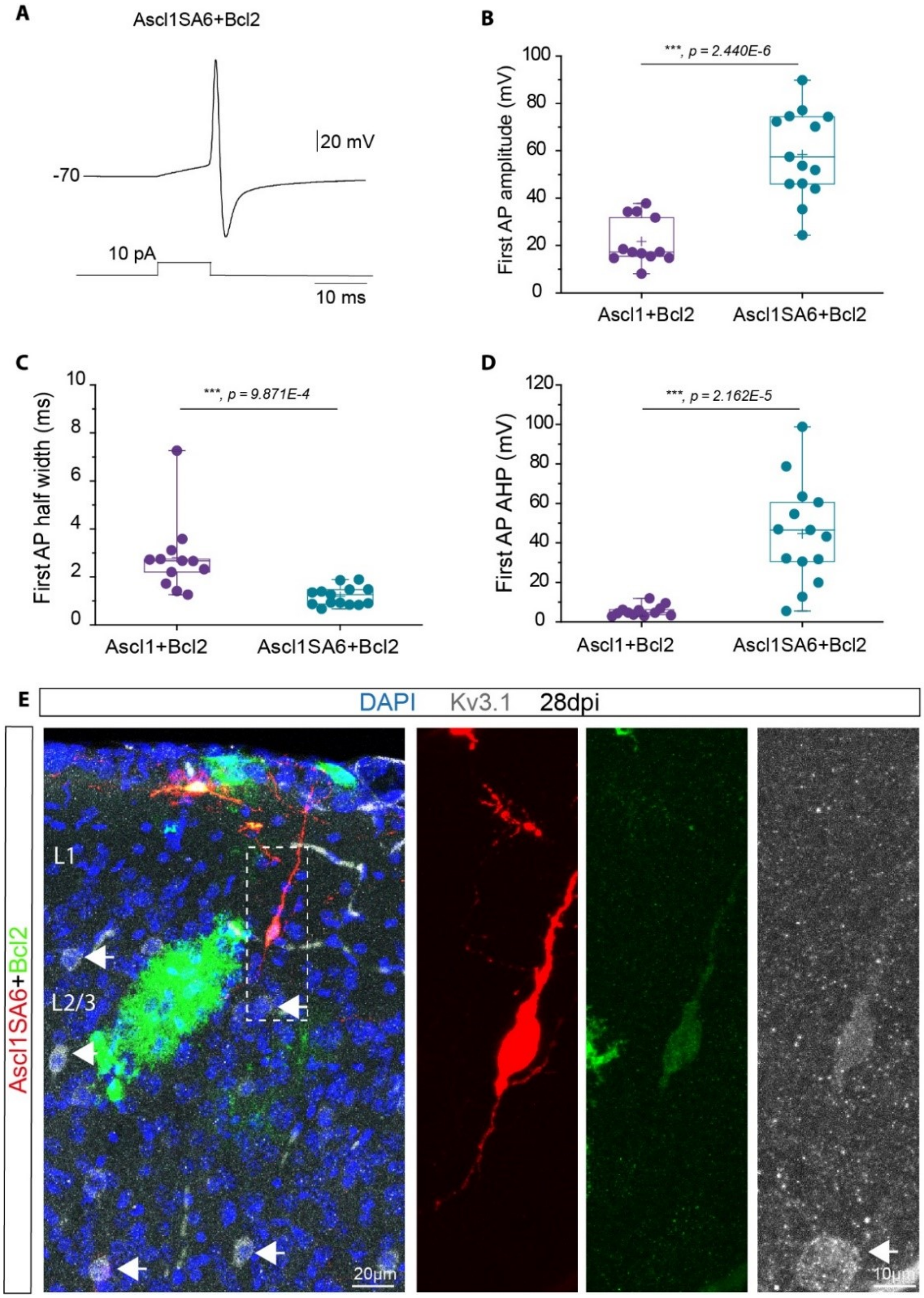

Fig. S11. Ascl1SA6/Bcl2 iNs exhibit a narrow action potential, a well-developed afterhyperpolarization, and express Kv3.1 (Related to Fig. 4). (A) Action potential recorded from an Ascl1SA6/Bcl2 iN after a brief depolarizing current pulse (10 ms), 28 dpi. (B-D) Action potential (AP) amplitude, AP half-width and AHP amplitude were measured for the first spike on recorded Ascl1/Bcl2 (12 cells, n = 5 mice) and Ascl1SA6/Bcl2 (14 cells, n = 8 mice) iNs at 28 dpi. Each dot represents one cell. Data shown with box and whisker plots, which give the mean (+), median, 25th and 75th percentiles and range. (E) Kv3.1 expression in an Ascl1SA6/Bcl2 iN. Note the high level of Kv3.1 in endogenous interneurons (arrows). High-magnification images correspond to the inset (white box). Two-tailed Student's unpaired t-test in (B-D).

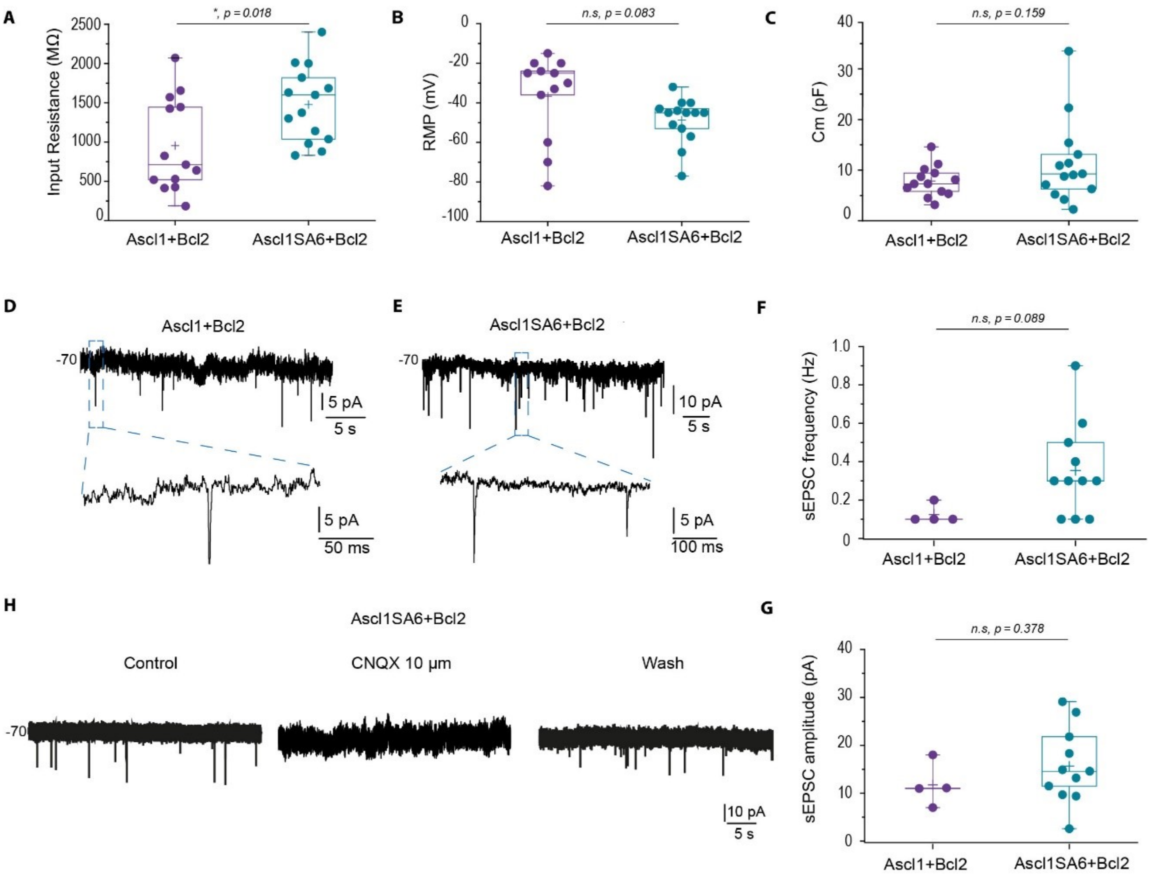

Fig. S12. The vast majority of Ascl1SA6/Bcl2 iNs receive excitatory inputs (Related to Fig. 4). (A-C) Input resistance, resting membrane potential (RMP), and membrane capacitance (Cm) of recorded Ascl1+Bcl2 (13 cells, n = 5 mice) and Ascl1SA6/Bcl2 (14 cells, n = 8 mice) iNs at 28 dpi. Each dot represents one cell. Data shown with box and whisker plots, which give the mean (+), median, 25th and 75th percentiles and range. (D-E) Spontaneous synaptic inputs recorded from an Ascl1/Bcl2 iN (D, 4 of 13 cells, n = 5 mice) or an Ascl1SA6/Bcl2 iN (E, 11 of 13 cells, n = 8 mice). Expanded presentations show individual events. (F-G) Frequency and amplitude of spontaneous excitatory postsynaptic currents (sEPSC) recorded from Ascl1/Bcl2 or Ascl1SA6/Bcl2 iNs. 4 out of 13 cells and 11 out of 14 recorded cells exhibited sEPSC and were therefore included in the analysis for Ascl1/Bcl2 and Ascl1SA6/Bcl2, respectively. Each dot represents one cell. Data shown with box and whisker plots, which give the mean (+), median, 25th and 75th percentiles and range. (H) Synaptic currents on Ascl1SA6/Bcl2 iNs were blocked by the AMPA/kainate receptor blocker CNQX and were recovered after the washout of the drug, confirming their excitatory nature (2 of 2 cells). Two-tailed Student's unpaired t-test in (A-C, F,G).
